# Tumor associated macrophages as key contributors and targets in current and future therapies for melanoma

**DOI:** 10.1080/1744666X.2024.2326626

**Published:** 2024-03-27

**Authors:** Shabana Habib, Gabriel Osborn, Zena Willsmore, Min Waye Chew, Sophie Jakubow, Amanda Fitzpatrick, Yin Wu, Khushboo Sinha, Hawys Lloyd-Hughes, Jenny L. C. Geh, Alastair D MacKenzie-Ross, Sean Whittaker, Victoria Sanz-Moreno, Katie E. Lacy, Sophia N Karagiannis, Rebecca Adams

**Affiliations:** a St. John’s Institute of Dermatology, School of Basic & Medical Biosciences, King’s College London, London, UK; b Oncology Department, Guy’s and St Thomas’ Hospital, London, UK; c Breast Cancer Now Research Unit, School of Cancer & Pharmaceutical Sciences, King’s College London, Innovation Hub, Guy’s Hospital, London, UK; d Peter Gorer Department of Immunobiology, School of Immunology & Microbial Sciences, King’s College London, London, UK; e St John’s Institute of Dermatology, Guy’s, King’s and St. Thomas’ Hospitals NHS Foundation Trust, London, England; f Department of Plastic Surgery, Guy’s, King’s and St. Thomas’ Hospitals, London, England; g The Breast Cancer Now Toby Robins Research Centre, Division of Breast Cancer Research, The Institute of Cancer Research, London

**Keywords:** Melanoma, macrophages, polarization, immunoregulatory, tumor microenvironment, immunotherapy, checkpoint inhibitors

## Abstract

**Introduction:**

Despite the success of immunotherapies for melanoma in recent years, there remains a significant proportion of patients who do not yet derive benefit from available treatments. Immunotherapies currently licensed for clinical use target the adaptive immune system, focussing on Tcell interactions and functions. However, the most prevalent immune cells within the tumor microenvironment (TME) of melanoma are macrophages, a diverse immune cell subset displaying high plasticity, to which no current therapies are yet directly targeted. Macrophages have been shown not only to activate the adaptive immune response, and enhance cancer cell killing, but, when influenced by factors within the TME of melanoma, these cells also promote melanoma tumorigenesis and metastasis.

**Areas Covered:**

We present a review of the most up-to-date literatureavailable on PubMed, focussing on studies from within the last 10 years. We also include data from ongoing and recent clinical trials targeting macrophages in melanoma listed on clinicaltrials.gov.

**Expert Opinion:**

Understanding the multifaceted role of macrophages in melanoma, including their interactions with immune and cancer cells, the influence of current therapies on macrophage phenotype and functions and how macrophages could be targeted with novel treatment approaches, are all critical for improving outcomes for patients with melanoma.

## 1. Introduction

Although it accounts for only 5% of skin cancers diagnosed, melanoma remains the deadliest cutaneous tumor type, in spite of the major advances in immunotherapy over the last decade. Added to this, melanoma diagnoses have increased in the last five years, with an incidence of 16,700 new melanoma skin cancers in the UK every year that is expected to continue increasing to reach 20,800 new cases per year between 2023–2025 [1,2]. Melanoma arises from melanocytes, the specialized pigmented cells found predominantly in the skin and eyes. A combination of genetic and environmental factors, such as chronic intermittent and prolonged UV radiation exposure, can result in sequential pathogenic mutations leading to constitutive activation of pro-survival signals in these cells and thus tumor growth [3]. Melanoma is considered the archetypal immunogenic tumor, with a tumor microenvironment (TME) consisting of a dense immune infiltrate. Interactions between melanoma cells and the immune compartment can be beneficial to tumor control, however, the same interaction can also promote the hallmarks of cancer, namely tumor cell invasion at the primary site, intravasation into the lymphatic system and the circulation, extravasation, and survival of tumor cells at distant sites [4].

Harnessing these immune interactions has been shown to be critical to increasing survival of patients with melanoma. Historically, advanced stage melanoma was resistant to conventional systemic therapies; however, in the last decade, novel immunotherapies and targeted therapies have transformed patient outcomes. Current standard of care immunotherapies predominantly work by blocking the regulatory functions of T cells, namely the cell-surface checkpoints programmed death-ligand1 PD-L1/PD-1 axis and the cytotoxic T-lymphocyte-associated protein 4 (CTLA-4). Small molecule inhibitors target mutant of BRAF (BRAFi) and MEK (MEKi) on the mitogen-activated protein kinase (MAPK) pathway to block cancer cell survival signals [5]. In early-stage disease (stage 0, I, II) surgery remains the preferred treatment with curative intent. For high-risk stage IIB and IIC disease, adjuvant immunotherapy has recently received regulatory approval [5]. In advanced unresectable disease settings (stage III and IV), immunotherapies and targeted therapies can extend survival, with evidence suggesting the former are more likely to yield durable/curative responses (https://ascopubs.org/doi/full/10.1200/JCO.22.01763). Despite clear advances in systemic therapies that have improved 5-year survival rates from 5% to 50%percent, survival rates remain poor in half of patients due to treatment resistance and toxicity, which can result in treatment discontinuation [6].

Melanoma is an immunogenic tumor whose relationship with immune cells resident in the TME significantly influences cancer cell proliferation, progression, and metastasis. Macrophages in the TME, referred to as tumor-associated macrophages (TAMs), are the most prevalent infiltrating immune cells, representing over 50% of the hematopoietic cell populations [7]. Yet, these cells are not the overt target of current therapies. While the presence of TAMs is often associated with poor prognosis, macrophages represent diverse populations with complex and multifaceted functional capabilities [7]. Emerging research suggests that macrophages are able to interact with current clinically approved therapies. Importantly, by manipulating their capacity for plasticity, these cells harbor significant potential to be directly targeted. In this review, we provide an overview of the origin, recruitment, and polarization of TAMs in the context of melanoma and insights into the multifaceted roles and therapeutic potential of TAMs.

## 2. Origins of tissue macrophages

Macrophages play essential roles in both the innate and adaptive immune responses, as well as in tissue homeostasis, repair, and immune regulation. Many theories have been postulated to categorize these highly plastic cells into different subsets and to decipher their roles within healthy tissue and in cancer.

For example, macrophages can be classified by their origins into monocyte-derived macrophages (MDMs) or tissue-resident macrophages (TRMs) [8]. In an adult, macrophages mainly originate from monocytes in the blood generated from myeloid progenitors within the bone marrow. Once recruited to different tissues, mainly via the CCL2/CCR2 pathway, monocytes then differentiate into macrophages depending on organ-specific cues [8]. TRMs are embryonic-derived, generated during earlier stages of ontogeny that persist throughout life and can be recruited to the TME via the CSF-1/CSF-1 R pathway [9]. TRMs are highly heterogenous, have self-renewal properties, and can be found in tissues such as the skin, eye, brain, and lung [9]. Within the context of cancer, although not yet clearly elucidated, the contribution of either TRMs or MDMs in tumors appears to be organ specific [10–12]. It has been suggested that the source of macrophages may vary depending on the stage of tumorigenesis and the cancer type [13]. In early stages of tumorigenesis, TRMs may predominate as the main infiltrating population, while in the later stages MDMs are recruited and polarized by the tumor, becoming the dominant TAM population [13].

Macrophages can be classified by function. For example, macrophages can have pro-inflammatory properties, contributing to immune defense via key mechanisms that include phagocytosis, antigen presentation and immunomodulation [14]. Upon recognition of pathogen-associated molecular patterns (PAMPs) or damage-associated molecular patterns (DAMPs) by cell surface pattern recognition receptors (PRRs), macrophages mount a rapid response to phagocytose target cells, such as pathogens or apoptotic cells [14]. Macrophages can then secrete cytokines to modulate the immune system and activate the complement cascade. Macrophages are also present engulfed exogenous antigens through the major histocompatibility complex class II (MHC II), and cross presentation on MHC I, to activate T helper cells via cognate T cell receptors, mounting an adaptive immune response [15]. Furthermore, macrophages may be involved in antibody dependent cellular cytotoxicity (ADCC), phagocytosis (ADCP) and complement activation (CDC) [14]. Several of these functions may also be seen in anti-tumor immunity and cancer surveillance [15].

On the other hand, macrophages can also exhibit immunoregulatory roles, evident in their ability to maintain tissue homeostasis and coordinate tissue repair. They can perform these juxtaposing mechanisms via an array of mediators to potentiate complex pro-inflammatory or anti-inflammatory cascades, depending on the local tissue conditions in which they operate [14].

Categorizing macrophages by function was historically, and perhaps simplistically, achieved using the M1/M2 spectrum [16]. The ‘classically activated’ M1 phenotype is typically described as pro-inflammatory, and in the context of cancer, thus tumoricidal, whilst the alternatively activated M2-like phenotype is considered anti-inflammatory, regulatory, immunosuppressive or pro-tumorigenic [16]. ‘M1-like,’ or classically activated macrophages are involved in antigen presentation and mediate intracellular pathogen or tumor cell destruction [16]. These cells typically express high levels of cytokines and chemokines such as IL-12, IL-6, CXCL10, IFN-γ, IL-23, and TNF-α, and other secreted mediators such as nitric oxide synthase (NOS) and reactive oxygen species (ROS) [16,17]. The production of ROS may also increase TNF-α and MCP-1 secretion, further contributing to enhanced infiltration of macrophages into tissues including into the TME [18]. On the other hand, ‘M2-like,’ or regulatory macrophages are characterized by high expression of cytokines such as IL-10 and TGF-β, which among other functions support expansion of regulatory T cells (Tregs) [16] and vascular endothelial growth factor (VEGF) which supports angiogenesis and inhibits T cell activity [19,20]. Together, these create an immunosuppressive environment amenable to tumor proliferation and metastasis (8). It is widely acknowledged that a binary M1 or M2 nomenclature represents only extreme phenotypes. Based on their extraordinary plasticity and diversity in morphology, cell surface, and intracellular markers, secretomes and functions, macrophage populations likely represent a wide spectrum of polarization phenotypes, continuously influenced and regulated by immune and inflammatory milieu [21]. This milieu may differ by cancer type, organ and the local tissue in which macrophages reside [22].

## 3. TAMs in the melanoma tumor microenvironment

### 3.1. Melanoma derived factors can influence macrophage polarisation

Within the continuum of diverse phenotypes of TAMs in the TME of melanoma, a process of adaptation from an immune activating to immunosuppressive and tumor-promoting functions may occur early [22]. Melanoma-derived soluble factors and metabolites, such as TGF-β, IL-10, IL-4, and IL-13 May contribute to this shift toward acquisition of pro-tumorigenic features [23]. Tumor-derived granulocyte-macrophage colony-stimulating factor (GM-CSF), has been reported to downregulate macrophage-mediated cytotoxic functions [24]. Melanoma exosomes have been shown to induce a spectrum of macrophage phenotypes secreting a variety of anti-inflammatory (IL-4, IL-10, IL-11, and IL-13) but also pro-inflammatory (TNF-α, IL-12B, IL-1β, IL-6, iNOS, and CCL22) factors, reflecting the spectrum of phenotypes detected in the TME [25]. Hypoxia can be another driver of macrophage polarization toward immunosuppressive phenotypes. In hypoxic environments, melanoma cells have been reported to enhance release of the DAMP High-Mobility Group Box 1 protein (HMGB1), driving accumulation of M2-like macrophages at the tumor site and release of IL-10 [26]. A recent study also showed that lactic acid, produced by tumor cells as a by-product of hypoxic glycolysis, induced VEGF expression, and M2-like polarization of TAMs, promoting tumor development [26].

### 3.2. TAMs interact with immune cells in the TME to promote both anti- and pro-tumour effects

#### 3.2.1. TAMs and T cells

Macrophage polarization states may also be influenced by a variety of interactions with different immune cells in the TME. The interaction between TAMs and T cells is reciprocal and complex. TAM polarization is known to be regulated by T cells, while macrophages also have a large influence on T cell activity [27].

For example, in the initial stages of tumorigenesis, TAMs play a pro-inflammatory role resulting in tumor suppression, with IFN-γ activating TAMs able to exert a killing effect on tumor cells [28]. As well as this, TAMs mediate the T cell responses by upregulating co-stimulatory molecules, such as CD80 and CD86, activating and recruiting CD8+ T cells via CD28 engagement, to initiate the release of chemokines and cytokines further promoting an inflammatory response [29].

Tumor progression is associated with an immunoregulatory-TAM phenotype switch, which creates a tumor-promoting environment through the expression of T cell immune checkpoint ligands on TAMs including PD-L1 inhibiting T cell activation [30]. TAMs are also able to influence T cell recruitment, demonstrated in studies in which depletion of TAMs is shown to restore T cell migration and infiltration into the TME, improving the efficacy of immune checkpoint inhibitors (ICIs) such as anti-PD1 immunotherapy [27].

The interaction between macrophages and Tregs may play a key role in tumor-associated immunosuppression [31]. Tregs promote macrophage polarization toward an immunoregulatory phenotype [31]. This is done through the secretion of cytokines such as IL-4, IL-10, TGF-β and IL-13 by Tregs [32]. Such macrophages in return secrete TGF- β and promote expression of PD1 on CD4+ T cells, resulting in further infiltration of Tregs into the tumor [32]. As well as this, Tregs inhibit the release of IFN-γ by CD8+ T cells, inhibiting the presence of tumor-killing macrophages in the TME [31].

**Figure 1.**
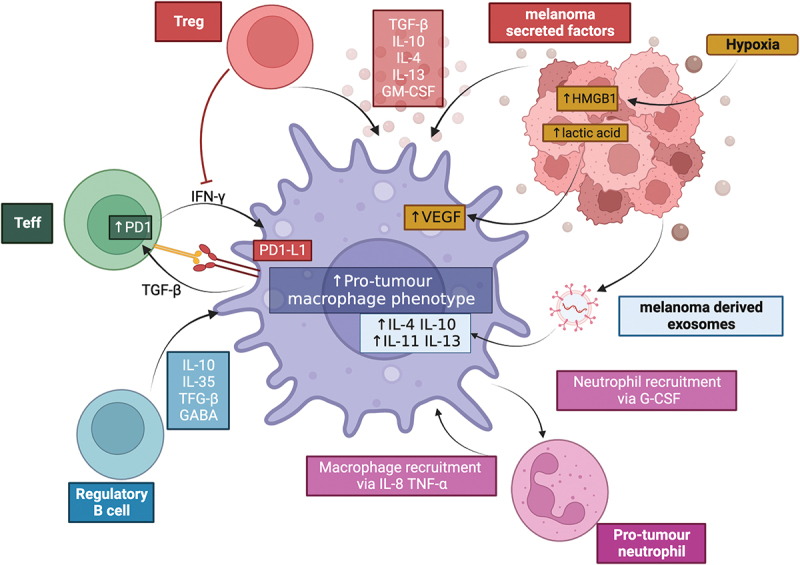
Macrophages can be polarised to a pro-tumour phenotype by cells in the TME. Macrophages can be influenced by melanoma-secreted factors, melanoma-derived exosomes and melanoma-derived products of hypoxia metabolism. Regulatory T cells (Treg) can reduce IFNγ production, required for pro-inflammatorymacrophage polarisation, as well as produce immunoregulatory cytokines including TGFF-β, IL-10 and IL-4. Effector T cells (Teff) and macrophages can interact via the PD-1/PD-L1 axis, reducing T cell activation and further promoting an immunoregulatory environment. B cells can secrete multiple immunoregulatory factors that influence macrophage states; and pro-tumour neutrophils reciprocally interact with macrophages to increase the recruitment and polarisation of both pro-tumour macrophages and neutrophils. Created with BioRender.com

Figure 1 summarizes the interaction between macrophages and cells within the TME to drive polarization to a pro-tumor phenotype.

### 3.3. Manipulating TAM-T cell interactions through cell surface receptors

#### 3.3.1. CD40

CD40, a co-stimulatory molecule, is strongly expressed on macrophages and binds to the CD40 ligand present on CD4+ T cells to activate T cell maturation functions via its interaction with its ligand CD40L on the T cell surface [33]. Agonistic CD40 monoclonal antibodies can stimulate the activation of pro-inflammatory marker genes, which in turn can reeducate TAMs toward an anti-tumorigenic phenotype [34]. CD40 monoclonal antibodies can also exert an effect through a T cell independent, macrophage-dependent anti-tumor mechanism. CD40 agonists bind CD40 expressed on macrophages resulting in the release of IFN-γ and CCL2, redirecting CCR2+ macrophages to infiltrate tumors until the CD40 signal ablates [35]. As well as this, CD40 activation triggers fatty acid oxidation (FAO) and glutamine metabolism, promoting epigenetic reprogramming of pro-inflammatory genes and an anti-tumor macrophage phenotype [33]. This dual mechanism of action of CD40 agonists, may therefore highlight a novel therapeutic option (see Table 1).

**Table 1.** Examples of current and ongoing clinical trials targeting macrophages in cancer therapy.

Drug Name	Type	Target	Cancer Type	Phase	Status	Route of Drug Administration	Outcome	Reference
Plozalizumab Vedolizumab	mAb – IgG1	CCR2	Melanoma	1b	Terminated	Intravenous	Protocol efficacy futility met	NCT02723006
Carlumab	mAb – IgG1	CCL2	Solid tumors	1	Completed	Intravenous	No objective anti-tumor response observed	NCT00537368
Sotigalimab	mAb – IgG1	CD40	Solid tumor	1b	Active	Intravenous		NCT03502330
CP-870,893	mAb – IgG2	CD40	Melanoma	1	Completed	Intravenous	Combination therapy shows clinical efficacy	NCT01103635
Cabiralizumab	mAb – IgG4	CSFR-1	Solid tumor	1b	Active	Intravenous		NCT03502330
Emactuzumab	mAb – IgG1	CSFR-1	Solid tumors	1/1b	Active	Intravenous		NCT01494688
Emactuzumab	mAb – IgG1	CSFR-1	Solid tumors	1	Completed	Intravenous	Manageable safety profile and considerable objective response rate was observed	NCT02323191
Poly-ICLC	dsRNA	TLR3	Solid tumor	1	Active	Intratumoral		NCT04116320
BO-112	dsRNA	TLR3	Melanoma	2	Active	Intratumoral		NCT04570332
Bevacizumab	mAb – IgG1	VEGF	Melanoma	2	Recruiting	Intravenous		NCT04356729
AT13148	Protein kinase inhibitor	ROCK-AKT	Solid tumor	1	Completed	Orally	No results published	NCT01585701
Vactosertib	Protein kinase inhibitor	TGF-β	Melanoma	2	Recruiting	Orally		NCT05436990
Panobinostat	Hydroxamic acid	HDAC	Melanoma	1	Completed	Orally	Results pointed against the use as monotherapy	NCT01065467
Panobinostat	Hydroxamic acid	HDAC	Melanoma	1	Completed	Orally	No additional benefit observed in response rate when combined with anti-CTLA4	NCT02032810
0508CT-	CAR-M	HER-2	Solid tumors	1	Recruiting	Intravenous		NCT04660929

#### 3.3.2. Marco

Macrophage receptor with collagenous structure (MARCO) is a scavenger receptor expressed almost exclusively on tumor-promoting TAMs and are thought to have an immunoregulatory role within the TME supporting tumor growth [36]. MARCO ligation to modified cell-antigens, such as apoptotic cells or tumor cells, induces activation of the MEK/ERK/p90RSK/CREB signaling cascade, promoting the release of IL-10 and upregulation of PD-L1 in a STAT3-dependent manner [37], which contributes to an immunosuppressive TME through enhancing Treg cell proliferation, blocking cytotoxic CD8+ T cell and NK cell activation [38]. Upregulation of surface MARCO expression can be induced by certain cytokines including IL-1a, IL-6 and IL-10 present within the TME [38].

Inhibition of MARCO can reverse the immunosuppressive effects of TAMs, which in turn has been shown to reduce tumor progression in murine models of solid tumors [39]. Upon inhibition of MARCO, macrophages release TNF-related apoptosis-inducing ligand (TRAIL) promoting natural killer (NK) cell-mediated killing and results in downmodulation of Tregs, reduction of IL-10, and reestablishes cytotoxic T cell activity [37]; Therefore, anti-MARCO antibody treatment used in combination with T cell-directed immunotherapy, such as antibodies to PD-1 or PD-L1 May provide a promising approach to combinatorial immunotherapy for melanoma.

#### 3.3.3. Sting

Stimulator of interferon genes (STING) is a type 1 interferon driver mediated by ligands such cyclic GMP-AMP (cGAMP) [40]. STING-triggered tumor-infiltrating macrophages showed more M1-like phenotype compared with other TAMs [40]. STING also exerts anti-tumor effects by promoting CD8+ T cell activity through upregulation of MHC class I molecules and NK cell infiltration [41]. Intratumoral injection of cGAMP resulted in accumulation of activated macrophages in the TME in a STING-dependent manner resulting in an antitumour immune response [40,41]. STING signaling contributed to the activation of the NOD-like receptor protein 3 (NLRP3) [42]. NLRP3 is a multiprotein complex that regulates macrophages, promoting secretion of pro-inflammatory cytokines such IL-18 and IL-1β by macrophages and this in turn was shown to optimize anti-tumor activity of NK cells and restrict tumor growth [42]. [42] Similarly, low levels of expression of STING in hepatocellular carcinoma cells has been associated with increased tumor volume and decreased CD8+ T cell infiltration [43]. Several STING agonists have shown potent anti-tumor efficacy with good tolerability in melanoma-bearing mice models and clinical applicability may therefore be promising [44].

### 3.4. TAMs and B cells

Macrophages may interact with regulatory B cells (Bregs). Transfer of B1a Bregs in mouse melanoma models has been shown to exacerbate tumor growth [45], a process thought to be mediated by the secretion of anti-inflammatory factors including IL-10, IL-35 and TGF-β by Bregs [46]. In the human setting, a study reported enhanced circulating TGF-β+PD-L1+ B cells and TGF-β+:TNF-α+ B cell ratios and lower pro-inflammatory TNF-α+ B cells and IFN-γ+:IL-4+ B cell ratios as a feature of melanoma [46]. TGF-β-expressing B cells were also detected in human melanoma lesions assembling in clusters [46]. These skewed B cell responses are expected to impact on macrophage phenotypes and their functions. TGF-β has been shown to transform macrophages into an immunosuppressive phenotype. IL-10 results in inhibition of macrophage function [47,48]. IL-10 also plays a key role in reducing the proportion of cytotoxic T cells further resulting in pro-tumor remodeling of the TME [45]. B cell-derived GABA signaling has also been implicated in promoting monocyte differentiation into anti-inflammatory macrophages with subsequent upregulation of secretion of IL-10 and inhibition of CD8+ T cell cytotoxic function [49]. Thus, by inactivating the B cell-specific GABA-generating enzyme GAD67, an anti-tumor response can be enhanced, offering a potential new therapeutic target [49].

Recent studies have also highlighted that murine, and possibly human cancers, may also stimulate transformation of B cell precursors into macrophage-like cells to generate immunosuppressive TAMs [50]. Mouse models of solid tumors have shown the differentiation of B cell precursors to generate macrophage-like cells (B-MFs) in response to tumor-secreted CSF that activate the CSF-1 R signaling pathway [50]. Unlike monocyte-derived macrophages, B-MFs are more efficient at phagocytosing apoptotic cells, suppressing proliferation of cytotoxic T cells and inducing a rise in Tregs [50]. This, in turn, promotes tumor progression and metastasis [50]. Even though myeloid-biased differentiation in the bone marrow is traditionally considered the primary source of TAMs, the differentiation of splenic hematopoietic stem, erythroid progenitor cells, and B precursor cells in the spleen, have all also been depicted as important origins of TAMs [51]. It may therefore be possible to utilize the heterogeneity and plasticity of TAMs as a future therapeutic target in the context of melanoma, by targeting tumor-promoting immune cells and reeducating them into TAMs with an anti-tumor phenotype.

### 3.5. TAMs and neutrophils

Neutrophils are phagocytic cells, which can be polarized into an anti-tumor N1 or pro-tumor N2 phenotype [52]. The presence of tumor-associated neutrophils has been correlated to poor prognosis in melanoma patients [53]. It is thought that melanoma cells reshape the TME by secreting various cytokines including TGF-β, IL-6, and IL-8 that trigger a pro-tumor N2 phenotype in neutrophils [54]. N2 polarized neutrophils, in turn, confer stem-like traits to melanoma cells, sustaining melanoma development and progression [54]. Through the secretion of IL-8 and TNF-α, neutrophils recruit macrophages to inflammatory environments; in turn, TAMs trigger a reciprocal signaling cascade involving IL-17 expression by T cells leading to G-CSF-induced migration of pro-tumor N2 neutrophils [52].

The interaction of macrophages within the wider immune system and the mechanisms driving early polarization that favor immune-activating phenotypes versus the signals that promote immunosuppressive signals in advanced disease are still being elucidated. Understanding this may help create immunotherapy approaches for melanoma by allowing targeting of specific signaling molecules and pathways.

### 3.6. Interaction of TAMs with myeloid-derived suppressor cells (MDSCs) and dendritic cells (DCs)

MDSCs are a group of heterogeneous bone marrow-derived immature myeloid cells associated with chronic inflammation and tumor progression. Their pro-tumoral roles are thought to be mainly mediated by suppressing CD8+ T cell and NK cell activation and cytotoxic activities [55] Veglia et al. 2021). Macrophages and MDSCs are present within most solid tumors, and contribute to the creation of an immunosuppressive TME and unfavorable prognosis [55]. MDSCs exert an immunosuppressive effect on macrophages by releasing IL-10, thus inhibiting macrophage expression of IL-12 and promoting M2-like-phenotype switching (Parker et al., 2014). These mechanisms impair anti-tumor immunity and promote tumor invasion and metastasis. A study in an inducible CSF1R knockout mouse model of melanoma demonstrated that granulocytic myeloid-derived suppressor cells (G-MDSCs) persist following CSF1R blockade which depleted TAMs (Banuelos et al. 2024). G-MDSCs furthermore suppressed macrophage phagocytosis, while CXCR2 blockade of G-MDSCs promoted macrophage pro-inflammatory phenotype and melanoma clearance (Banuelos et al. 2024).

Dendritic cells (DCs) can exert both an anti-tumor and pro-tumor functions. They can initiate an anti-tumor immune response by cross-presenting tumor-associated antigens predominantly to CD8+ T cells [56]. Cross-presenting DCs may interact with antigen-presenting macrophages to activate cytotoxic T cells [57]. This process is further enhanced by the production of IFN-I derived from macrophages and therefore plays a key role in promoting anti-tumor effects [57]. Melanoma cells can exploit DC versality to subvert their functions toward pro-tumor phenotypes that promote Tregs and a Th2 response [58]. Interestingly, tumor-infiltrating CDs have been shown to express high levels of markers characteristic of TAMs, such as CD206 and CD163, suggesting these cells exhibit a TAM-like pro-tumoral function [59].

MDSCs can migrate to different peripheral organs and rapidly differentiate into mature macrophages and DCs [60]. This highlights likely interlinked roles between MDSCs, DCs, and macrophages in driving tumor growth.

### 3.7. Tumour-promoting functions of TAMs in melanoma

Melanoma progression and prognosis correlates with increasing abundance of TAMs and a shift in their phenotypes within the TME [21]. This reflects a multitude of attributes with which these cells may support melanoma progression and the process of metastasis. TAMs secrete chemotactic factors, such as CCL2 and CCL1, which promote further macrophage recruitment and polarization, as well as migration of other cells, such as tumor-promoting neutrophils, to support an immunosuppressive TME [61,62]. TAMs are reported to feature reduced cytotoxic functions, poor antigen-presenting ability, and expression of the checkpoint molecule PD-1; all of which have been correlated with disease progression and poor therapy response [63,64].

Macrophages can contribute to angiogenesis [65–67]. The secretion of TNF-α and IL-1α by activated TAMs triggers the production of IL-8 and VEGF-A by melanoma cells, resulting in enhanced angiogenesis and leakier vasculature [68]. Together with IL-4 and IL-10, VEGF-A also acts as a chemoattractant for immunosuppressive macrophages [69]. Additionally, melanoma polarizes immunoregulatory macrophage phenotypes able to secrete adrenomedullin (ADM), a potent and long-lasting vasodilator, as well as upregulates its receptors to promote pro-angiogenic and cancer cell proliferation processes by improving the blood supply to tumor cells [70]. Furthermore, expression of the macrophage inhibitory cytokine-1 (MIC-1), a member of the TGF-β family regulated by mutant BRAF melanomas, is enhanced in the TME and the patient circulation [71]. MIC-1 is a cytokine which regulates various cancer hallmarks including proliferation and promotion of a pro-tumor inflammatory environment, inducing invasion, metastasis, angiogenesis, and resisting cell-death [71,72].

TAMs can play a key role in promoting a metastatic cascade of cancer cells within melanoma [2]. The epithelial–mesenchymal transition (EMT) is a complex developmental process that morphologically transforms tumor cells to allow loss of cell–cell junctions and detachment from the basement membrane, enabling migration into the surrounding stroma and formation of distant metastasis [73], a process known as phenotype-switching in melanoma. Several studies in other cancer types have shown that TAMs regulate the EMT-like process to promote metastasis, through the secretion of IL-8, TNF-α and TGF-β allowing downregulation of E-cadherins, required for maintenance of cell–cell junctions [74–76].

Macrophages are found to be localized to blood vessels within the TME and assist in intravasation of tumor cells into the circulation [77]. Macrophages within the TME release proteolytic enzymes that breakdown the extracellular matrix [78] and trigger a positive feedback loop consisting of tumor cell-produced CSF-1 and TAM-produced EGF promoting chemotactic migration of tumor cells toward blood vessels [79]. The Rho-kinase (ROCK) – myosin II pathway is a key regulator of invasive and metastatic behavior [80], driving contractile forces of tumor cells required for migration, metastatic colonization, and aggressive invasion [80,81]. Analysis of human melanoma biopsies demonstrated high myosin II activity in the invasive segments of tumors in proximity of CD206+CD163+ TAMs and vessels [82]. Melanoma cells secrete a complex set of proteins controlled by IL1α-NF-κB, and this in turn triggers a positive feedback loop with ROCK-Myosin II that enables recruitment of monocytes and their differentiation into tumor-promoting macrophages [82]. The melanoma-associated macrophages subsequently support melanoma cell growth, by secreting factors that support MAPK-ROCK2-Myosin II-dependent growth [83] Inhibition of ROCK-myosin II reduces the presence of pro-tumorigenic macrophages in the TME, contributing to tumor regression [82]. The combination of ROCK-inhibitors with anti-PD1 therapy, has also been shown to reduce PD-L1 expression on both tumor cells and macrophages, thus improving therapeutic efficacy [84].

Macrophages have been shown to assist tumor cells attach and extrude through vessel walls, and loss of macrophages has therefore been shown to dramatically decline the extravasation rate with a co-incidence failure of metastasis [85]. Studies have shown that there are two distinct types of macrophages that infiltrate melanoma metastasis, and the profile of these cells depends on their location [86]. iCD14+ macrophages are close to the tumor nests and T cells, with a primary role of presenting intracellular indigested tumor antigens. On the other hand, sCD14+ macrophages localize in the stroma, and these often correlate with better survival for melanoma patients [86]. Interestingly, Martinek et al. also demonstrated that the macrophage profile at different metastatic sites is homogeneous, suggesting that the cellular profile is shaped more by the neighboring melanoma cells rather than intrinsic properties of the metastatic tissue site [86]. It may therefore be important to take into consideration the topology of macrophages in metastatic disease when developing macrophage-targeted therapies.

Through a better understanding of the conditions that influence macrophages and how these cells respond to shape the TME, it may be possible to derive strategies to enhance immune-activating subsets and re-activate or deplete immunoregulatory and suppressive phenotypes.

## 4. Macrophages are active players in checkpoint inhibitor functions and patient outcomes

### 4.1. Mechanisms of action of checkpoint inhibitor antibodies

Monoclonal antibody ICIs are now first line treatment for advanced melanoma. These include inhibiting the programmed cell death-protein 1 (PD-1), its ligand programmed death-ligand 1 (PD-L1), and cytotoxic T-lymphocyte-associated antigen 4 (CTLA-4). CTLA-4, expressed on activated memory T cells and upregulated on FoxP3+ Tregs, competes with CD28 on the T cell for recognition of co-stimulatory CD80/86 on antigen presenting cells [87]. This delivers negative signals to moderate T cell activation [87]. A negative regulator of mature T cell functions, PD-1 is expressed on activated and exhausted T cells, B cells, NK cells, and in peripheral tissues. Its ligand PD-L1, whose expression is induced by inflammatory cytokines, such as IFN-γ, is found on immune cells including T cells, B cells, DCs, macrophages but also on cancer cells or cancer associated stromal cells [88]. PD-1-PD-L1 attenuates activation of antigen-educated T cells, limiting their functions and promoting immune tolerance [89].

ICIs include Ipilimumab, an IgG1 antibody specific for CTLA-4, Pembrolizumab and Nivolumab, IgG4 antibodies specific for PD-1, and Atezolizumab, an IgG1 antibody recognizing PD-L1 and many others. ICIs reverse inhibitory immune checkpoints to unleash effective T cell responses that can restrict tumor progression [90]. Although the response rates to ICIs in patients with melanoma is improving, around half of melanoma patients still do not respond combination anti-PD-1/anti-CTLA-4 therapies, whilst single agent response rates are even lower at ~ 30–40% for anti-PD-1 agents and ~ 10–15% for anti-CTLA-4 agents [91–93].

### 4.2. Macrophage Fc-receptor engagement: influencing ICI therapy outcomes

Although ICIs primarily target T cell mediated responses to activate a broad T cell response against the tumor, as a highly abundant and diverse immune cell population in the TME, macrophages are emerging as key players in influencing ICI functions via a range of mechanisms. Dissecting these attributes may help predict or delineate disparate therapy response outcomes.

ICIs used in melanoma are monoclonal antibodies (mAbs). As such, ICIs are composed of the fragment for antigen binding (Fab) region, the proportion of the antibody that binds target antigens, and the crystallizable fragment (Fc) region, which binds Fc receptors (FcR) on immune cells, including macrophages [94]. Macrophages express multiple FcRs, which when engaged by antibodies via the Fc region, can trigger a pro- or anti-tumor response [95]. Macrophages express activatory FcRs, including FcyRIIA, which leads to strong ADCC and ADCP activity against tumor cells when bound to IgG1 antibody isotypes (e.g. ipilimumab) [96]. In addition, activation of the ADCP cascade may allow macrophages to present tumor antigens to T cells in the TME, eliciting a long-lasting tumor-specific adaptive immune response [94]. Alongside, macrophages express FcγRIIB, which is the only inhibitory FcyR, transducing an inhibitory signal via ITIM domain signaling upon binding of antigen-bound IgG. This is known to reduce the capacity of mAbs to induce ADCC and ADCP [94].

Designing ICIs with an optimal Fc portion should take into account several considerations [94]. For example, anti-PD-1 mAbs including Nivolumab and Pembrolizumab are of the IgG4 subclass, which is the least immune activating isotype, with well-described impaired Fc-mediated effector functions [97,98]. IgG4 antibodies, however, retain affinity for FcγRI expressed on macrophages, meaning that they can still bind to and be sequestered by macrophages [94]. Time-lapse intravital imaging studies in mouse models of anti-PD-1 responsive cancers, have shown that macrophages uptake anti-PD-1 mAbs from the surface of T cells [99], reducing their efficacy, an unhelpful consequence of mAbs binding the high affinity FcRs on these macrophages. In addition, anti-PD-1 mAbs when bound to the FcRs of macrophages via the Fc portion and target T cells via the Fab, can promote T cell mediated cytotoxic killing by macrophages, reducing therapeutic efficacy [94,100]. Furthermore, IgG4 binding to FcγRs can not only impair PD-1 targeting but also the recognition of the inhibitory FcγRIIB could restrict pro-inflammatory macrophage properties via downstream ITIM signaling.

In contrast to the sometimes-unhelpful interactions between PD-1/PD-L1 mAbs and macrophages in the TME, the efficacy of anti-CTLA-4 mAbs may be enhanced by macrophages. CTLA-4 is highly expressed by tumor infiltrating Tregs. Aside from the Fab portion of Ipilimumab binding and preventing the regulatory action of CTLA-4 on T cells, Tregs expressing high levels of CTLA-4 could be targeted by ipilimumab with the Fc portion of the antibody binding FcγRs expressed on immune cells including macrophages, inducing ADCC of Tregs [101,102]. Genetic Treg ablation in mouse models has shown that the immune regulatory impact of anti-CTLA-4 mAbs may not be solely driven by their effect on T cell stimulaton, but also through engagement of FcγR on macrophages [103]. FcγR engagement by anti-CTLA-4 mAbs, induces rapid remodeling of the innate immune landscape through downstream activation of type 1 interferon signaling and reduction of suppressive macrophages; this likely contributes to the induction of a successful response as part of anti-CTLA-4 therapy [103].

Aside from engaging their FcRs, macrophages may be directly targeted by the Fab portion of ICIs. It has been shown that TAMs in melanoma tumor express PD-L1, which can regulate macrophage activation and proliferation [63,104]. Although not designed to specifically effect macrophage function, studies in mouse models of cancer have shown that anti-PD-1/PD-L1 blockage in vivo increases macrophage phagocytosis with subsequent reduction in tumor growth and prolonged survival [63]. Real-time imaging in whole tumor tissues has demonstrated accumulation of anti-PD-L1 mAbs in tumor tissues regardless of PD-L1 expression by tumor cells [105]. The response to checkpoint inhibitors was predominantly mediated through interactions of anti-PD-L1 antibodies with PD-L1 on host myeloid cells, which promotes T cell activation to mount an anti-tumor response [105]. This would explain why some patients with PD-L1 negative tumors also respond to PD-L1 blockade therapy.

### 4.3. Recruitment of TAMs and TAM-mediated recruitment of other effector cells

The effect of ICIs could be enhanced by altering the composition of the TME. For example, CSF-1 is a primary growth factor that controls recruitment, proliferation, and survival of macrophages through engagement with and activation of the CSF-1 receptor (CSF-1 R) [106]. Myeloid cell-rich tumors, require CSF-1 for TAM accumulation and maintenance. Blockade of CSF-1-CSF-1 R can functionally reprogramme macrophage responses, enhancing their antigen-presenting abilities to produce anti-tumor T cell responses [107]. F [107,108]

A phase 1b study (NCT02323191) evaluated the safety and efficacy of the CSF-1 R blocking mAb emactuzumab in combination with the anti-PD-L1 mAb atezolizumab in patients with advanced solid tumors [109]. Combination treatment demonstrated a manageable safety profile with an objective response rate of 5.6% in melanoma patients [109]. An increase of CD8+ TILs was also observed in patients who lacked TAM depletion and was associated with a clinical benefit [109]. It is speculated that this may be mediated by PD-L1 and CSF-1 R mAbs binding to persisting TAMs to influence downstream signaling and ultimately drive TAM reprogramming [109]. TAMs may be reprogrammed to have a lower dependence on CSF-1 as a survival signal [109], show emactuzumab-induced pro-inflammatory type I IFN release [110], or atezolizumab-induced PD-L1-dependent M1 TAM polarization [111,112]. An alternative treatment approach may include blockade of CCL2 signaling involved in recruitment of TAMs to the TME as discussed above, which was shown to reduce tumor progression in several experimental tumor models [113]. However, when taken into clinical trials, plozalizumab, an IgG1 antibody against CCR2, failed to show any discernible benefit (NCT02723006) (see Table 1).

#### 4.3.1. Enhancing T cell recruitment

In a large proportion of cancer patients, CD8+ T cells are excluded from the vicinity of cancer islets. This contributes to limited therapeutic response and poor clinical outcomes [114]. TAMs are an important determinant of T cell exclusion [114]. Macrophages form long-lasting interactions with CD8+ T cells, and in mouse tumor models depletion of macrophages, using CSF-1 R inhibitors, results in CD8+ T cell migration and infiltration into tumor islets [114]. When combined with anti-PD-L1 therapy, this further enhances CD8+ T cell migration subsequently delaying tumor progression [114]. Furthermore, following CSF-1 R inhibition, an increase in CCL2, CXCL9, and CXCL10 was also noted [114], increasing recruitment of myeloid cells through the CCL2/CCR2 axis [115], and cytotoxic T cells, NK cells, and macrophages via the CXCL9–10/CXCR3 axis [116]. Cytotoxic T cells and NK cells further release IFN-y, promoting polarization of macrophages to a pro-inflammatory phenotype [116]. These altered chemotactic mediators in the TME and the subsequent influx of pro-inflammatory cells demonstrate the profound effect of the depletion of anti-inflammatory macrophages and polarization to a pro-inflammatory phenotype can have on the whole TME [114].

An upregulation of CXCR3 ligands, CXCL9 and CXL10, in an IFNγ-dependent manner has also been demonstrated following dual PD-1/CTLA-4 blockade with subsequent improvement in therapeutic efficacy [117]. Single-cell RNA-sequencing analysis of patient tumor-infiltrating lymphocytes revealed that CXCR3 was expressed by CD8+ and CD4+ T cells, whilst CXCL9 and CXCL10 were predominantly expressed by macrophages following dual ICI treatment [117]. Depletion of macrophages halted CD8+ T cell infiltration, but not migration of CD4+ Tregs, and reduced therapeutic efficacy to dual ICI therapy [117]. This suggests that CD8+ T cells infiltrate the tumor via the CXCL9/CXCL10-CXCR3 pathway and therefore T cell expression of CXCR3 is cruicial for their migration toward CXCL9/10 produced by TAMs. Adding to the importance of this macrophage-T cell interaction, CXCL9/10 production was demonstrated to be dependent on the release of IFNγ and TNF-α produced by T cells [117]. As well as this, given that macrophages also express CXCR3, a positive feedback loop may be stimulated by CXCL9/10, further promoting both macrophage and CD8+ T cell recruitment [117]. This highlights a novel strategy to enhance CXCL9/10 production, particularly by macrophages, to enhance efficacy of immunotherapy in cancer patients.

TGF-β has also been shown to impair anti-PD-1/PD-L1 immunotherapy efficacy by inducing an upregulation of PD-L1 by tumor cells [118]. Macrophages release cytokines, such as TGF-β, causing T cell dysfunction, accumulation of Tregs and reduced infiltration of CD8+ T cells [119]. Combination therapy of TGF-β inhibitors and anti-PD-1/PD-L1 has been shown to restore cytotoxic activity of T cells and anti-tumor activity of anti-PD-L1 in mouse models of melanoma and is currently being explored in phase 1 trials (NCT05436990) (see Table 1) [120,121].

#### 4.3.2. Polarisation of TAMs towards pro-inflammatory phenotypes

Another strategy to enhance the effect of ICIs is to polarize macrophages into a more pro-inflammatory phenotype with the aim of enabling improved antigen presentation to, and activation of, T cells. Agonists to CD40 (CD40a) can trigger pro-inflammatory cytokine secretion and promote maturation and activation of macrophages and other antigen presenting cells to enhance anti-tumor activity, stimulate antigen presentation to T cells and promote influx of cytotoxic T cells [122]. A combination of CSF-1 R and CD40a may simultaneously augment T cell infiltration and alter TAM composition. Consequently, a phase 1 clinical trial (NCT03502330) investigated the efficacy of the treatment combination of a CD40 agonist (APX005M, sotigalimab) with a CSF-1 R inhibitor (cabiralizumab), offering a dual targeting approach to activate macrophages [122] (see Table 1). This combination alongside nivolumab showed good tolerability and signs of efficacy with upregulation of pro-inflammatory cytokines in solid tumors including melanoma [122]. However, optimization of the dosing frequency is required to assess wider clinical applicability in difficult-to-treat patient populations with limited therapeutic options.

MicroRNAs are a class of non-coding RNAs that regulate immune cells through the repression of certain genes [123]. miR-155 plays an expanding role in modulating the expression of CD8+ T cell effector genes such as IFNγ and TNF-α, within the TME [123]. Lack of miR-155 in T cells results in reduced IFN-γ expression by T cells; this decreases IFN-inducible gene expression by TAMs and subsequent reduction in the number of M1 macrophages, influencing the TME to promote anti-tumor immunity [123]. Combination treatment with miR-155 and ICI therapy may function via overlapping pathways; miR-155 expression within T cells allowed recovery of anti-tumor immune response by ICIs, and ICI treatment resulted in decreased expression of the targets repressed in miR-155 tumor-infiltrating T cells [123].

**Figure 2.**
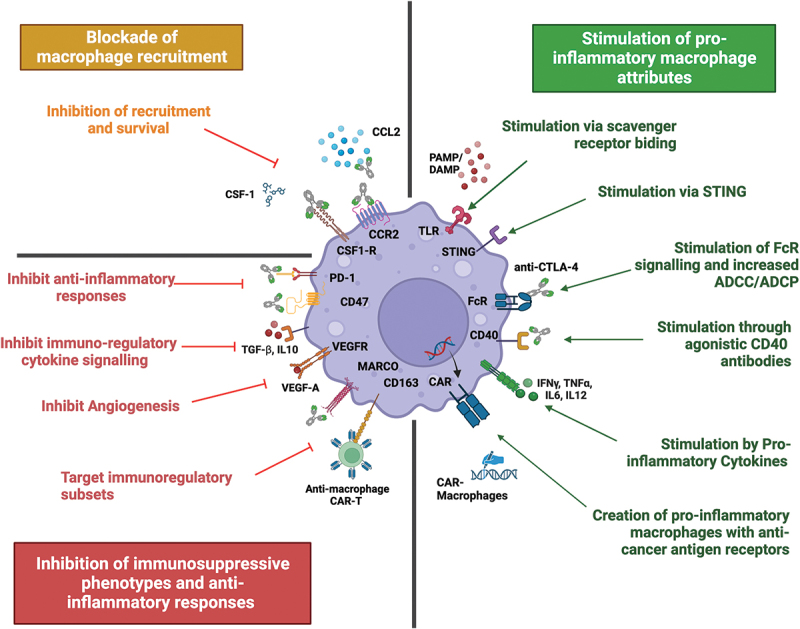
Harnessing macrophages to enhance current and future therapies. Macrophages can be harnessed to hone an anti-tumour immune response via several therapeutic strategies. The left side summarises approaches to inhibit macrophages and their pro-tumour functions. Top left: treatment approaches blocking recruitment and survival of macrophages. Bottom left: strategies focusing on inhibiting immunosuppressive functions of macrophages and re-educating TAMs into anti-tumour effectors. The right side summarises current treatment approaches that stimulate a pro-inflammatory TAM response, including stimulation through multiple cell-surface receptors and the creation of CAR-M with enhancing cancer killing abilities. Created with BioRender.com

In conclusion, this section demonstrates that macrophages play a key role in influencing the function of checkpoint inhibitors. Macrophages can be harnessed to hone an anti-tumor response using several novel therapeutic strategies, and when combined with current ICI treatments this can improve patient outcomes (Figure 2).

## 5. Involvement of macrophages in melanoma pathways and pathway inhibitor mechanisms: opportunities for therapy

Targeted therapies developed for melanoma include MAPK inhibitors, BRAFi and MEKi, These target the pathogenic constitutively activated MAPK pathway in BRAF- and NRAS- mutant melanoma cells [124]. Mutations in BRAF promoting cell proliferation and inhibiting apoptosis are present in approximately 50% of the cutaneous melanomas [125]. Inhibition of BRAF therefore halts tumorigenesis, a result which is significantly improved in combination treatments of BRAFi and MEKi therapies. While targeted therapies have improved progression-free survival and overall survival compared to historical treatments including chemotherapy, they are only effective in a subset of patients with specific mutations and the durability of response is often limited due to the development of treatment resistance [126].

TAMs often support neoplastic growth through their contribution to development of treatment resistance [127]. In vivo BRAF-mutant melanoma models with fluorescent markers to track stage-specific changes in macrophages under targeted therapy with BRAFi/MEKi, showed a rise in CCR2+ macrophage infiltration at the onset of a drug-tolerant persister (DTP) state after several weeks of treatment [127]. The DTP state refers to tumor cells entering a reversible slow proliferation state, which allows tumor cells to survive drug therapy long enough to acquire mechanisms of drug-resistance [128]. This can therefore have considerable clinical implications on response rates to targeted therapies. Various potential mechanisms have been revealed to mediate survival of DTP cells, including slow or no proliferation, adaptive energy depletion, microenvironmental adjustments and phenotypic plasticity [129]. All these mechanisms are thought to be related to the redox signaling pathway, whereby DTP cells undergo metabolic reprogramming to promote mitochondrial oxidative respiration and an antioxidant process [129]. A therapeutic strategy may therefore be to increase intracellular ROS levels by using pro-oxidants such as transition metals or free fatty acids to interfere with the antioxidant functions of DTPs, thereby causing cell death [129]. On the other hand, a CCR2-deficient TME delayed the onset of resistance and shifted melanoma cells to a more sensitive state to targeted therapy [127]. Importantly, this phenotype was reversed with the introduction of CCR2+ macrophages in the TME of melanoma [127]. In another study, overexpression of CSF-1 R was demonstrated in patients with BRAF and other MAPK activating mutations [130]. As previously mentioned, CSF-1 R is expressed on TAMs and, enabling their recruitment toward CSF-1 ligand, which also activates the ERK and PI3L/AKT pathways stimulating proliferation, differentiation, and survival of macrophages [130]. BRAF inhibition can result in rebound ERK activation with an associated upregulation of CSF-1 R and its ligand [130]. Inhibition of CSF-1 R inhibits rebound of ERK in BRAF-treated melanoma, shifting the TME toward a pro-inflammatory state with subsequent reduction in growth and invasiveness of melanoma cells [130]. These effects were further improved with the synergistic use of coinhibition of CSF-1 R and BRAF [130].

Melanoma cells expressing vascular endothelial growth factor receptor 1 (VEGFR-1) particularly demonstrate resistance to this therapy [131]. Vascular endothelial growth factor A (VEGF-A), a proangiogenic signal, interacts with VEGFR-1 to promote a tumorigenic milieu contributing to angiogenesis and TAM recruitment [131,132]. Targeting VEGFR-1 has been demonstrated to decrease macrophage recruitment [131]. In the setting of BRAFi resistance, it is recognized that BRAFi can lead to paradoxical activation of the MAPK pathway in non-mutant cells in the TME, including in TAMs, driving these cells to an immunosuppressive VEGF-secreting state, which in turn reactivates the MAPK pathway in melanoma cells [131]. Thus, blockade of VEGF and its interaction with VEGFR-1, in conjunction with the use of BRAFi may provide a potential treatment approach (see Table 1)

Given the reported roles of TAMs in BRAF mutant melanoma influencing tumor progression and response to targeted treatments, altering the macrophage protumour function may present a potential therapeutic strategy to improve melanoma treatments [133,134].

## 6. The role of macrophages in oncolytic virus therapies used in melanoma

Talimogene laherparepvec (T-VEC) is an intralesional oncolytic viral immunotherapy, consisting of a genetically modified oncolytic herpes simplex virus, approved for the localized treatment of advanced unresectable melanoma. The phase III study Oncovex Pivotal Trial in Melanoma (OPTiM) demonstrated a durable response over 6 months in patients in the T-VEC arm with improving overall survival in metastatic melanoma [135,136]. T-VEC’s tolerable side-effect profile also favors its use; however, since it is administered intralesionally, its use is limited to accessible metastasis and patients with visceral metastases do not benefit [135,136].

Early pre-clinical studies in melanoma reported anti-tumor effects of T-VEC through the direct infection and destruction of cancer cells [137]. However, recent studies have demonstrated an insight into the immunological functions of T-VEC [138–140]. Oncolytic viruses (OV) eliminate cancer cells by activating apoptotic pathways, creating pathogen-associated molecular pattern molecules, PAMPs [137]. PAMPs are a diverse set of microbial molecules that share patterns, which bind to pattern recognition receptors (PRRs) on immune cells including dendritic cells, NK cells and macrophages to stimulate their activation and migration [137]. These immune cells then gather tumor antigens and present them to T cells to further trigger a durable, adaptive immune response [137].

Toll-like receptors (TLRs), are one example of PRRs, expressed on immune effector cells. TLRs play an important role in triggering an early immune response and in polarizing macrophages to a pro-inflammatory phenotype [141]. TLRs differ in their cell expression profile, intracellular signaling pathways, and subsequent influence on the TME [142]. For example, TLR3 agonists have been shown to polarize macrophages toward an immune-activating phenotype in preclinical models of melanoma, and this increases the macrophage antigen presenting capabilities and decreases expression of PD-L1 and inhibitor receptors on infiltrating monocytes via the type 1 interferon signaling pathway [142]. In contrast, TLR7 and TLR9 agonists are potent drivers of anti-tumor macrophages, and these macrophages in turn secrete pro-inflammatory cytokines including IL-12 and type 1 interferon to activate CD8+T cells highlighting a therapeutic potential to use different TLR agonists to cause a synergistic effect [142]. Some studies have reported that melanoma cells can block macrophage activation through the suppression of TLR signaling [142]. Given that TVEC can increase the presence of PAMPs within the TME, further enhancing TLR stimulation by the inclusion of TLR ligands, may further promote macrophage polarization and subsequently enhance their tumoricidal effects of TVEC. Clinical trials evaluating intratumoral TLR agonists in combination with immunotherapy include NCT04116320 and NCT04570332 (see Table 1).

Initially, macrophages can have a barrier function that limits the efficacy of the OV [143]. Complement proteins bind the OV particles to opsonise them, allowing recognition and phagocytosis by M1 macrophages [144]. Macrophages also phagocytose infected tumor cells and present viral antigens to T cells for an adaptive immune response [144]. This suggest that anti-cancer activity of OVs may be impaired by a macrophage-mediated response, however, it is important to carefully evaluate the studies suggesting an obstructive macrophage response to OVs, as the majority of the data have been obtained from murine models limiting its translational application to humans [145]. To prevent macrophage-mediated removal of OVs, it may be possible to redirect TAMs toward tumor cells for therapeutic benefits. Cao et al. engineered an OV vaccine that disrupted the CD47/SIRPa [146]. CD47 is a ‘don’t eat me’ signal overexpressed on tumor cells and this interacts with its ligand SIRPa, which is a protein expressed on macrophages and dendritic cells, to prevent phagocytosis [146]. Blockade of the CD47/SIRPa signaling pathway enables macrophage phagocytosis of tumor cells that were otherwise protected [146]. Genetically modified SIRPa-Fc can block CD47, and these can therefore be delivered by OVs to hone an immune response toward tumor cells by macrophages exerting a therapeutic benefit [146].

Another strategy to enhance TVEC efficacy is by using it in combination with histone deacetylases inhibitors, HDACi, to enhance oncolysis and anti-tumor immunity [138]. HDACs are enzymes that interact with a wide spectrum of substrate proteins, involved in a range of cellular processes including exertion of antitumor activity through its effects on immune and survival-related pathways [147]. HDAC6 plays a key role as an immune checkpoint regulator in human melanoma cells [147]. Inhibition has been shown to exert an anti-tumor effect, however, only in immunocompetent mice, suggesting that the anti-tumor activity of HDAC6 inhibitors (HDAC6i) requires an intact adaptive immune system [147]. When OVs were combined with HDACi an anti-melanoma response was observed via monocyte-mediated type I IFN production that activated NK cells and lead to viral maturation of dendritic cells to potent antigen-presenting cells for cytotoxic T cell priming [138]. The addition of HDACi to OVs also lead to increased viral replication within tumor cells with subsequent enhanced oncolysis and anti-tumor immunity [138].

## 7. Harnessing macrophages and their mechanisms in novel treatments

Advances in understanding of the role of TAMs in the TME have allowed research into the development of numerous novel strategies designed to alter the pro-tumor functions of macrophages. This can be done through mechanisms including blockade or depletion of TAM recruitment, activating polarization to an anti-tumor phenotype, and adapting TAM-based communications among the TME (Figure 2).

### 7.1. Macrophage FcRs can be engaged by anti-cancer-antigen monoclonal antibodies

A growing field within oncoimmunology is the design of mAbs against cancer-specific antigens. Within the context of melanoma, creating a mAb with a Fab region against a melanoma-restricted antigen, and a Fc portion optimized to activate TAMs and increase their ability to perform ADCC and ADCP, could be highly effective, allowing the activation of TAMS localized to cancer cells, promoting their repolarization and the subsequent creation of an inflammatory TME [148]. One such antigen currently under investigation is chondroitin sulfate proteoglycan 4 (CSPG4), expressed in 70% of melanoma but demonstrating low expression in healthy tissue [149]. In human xenograft models of melanoma, an anti-CSPG4 IgE has been shown to restrict tumor growth and prolong the survival of xenograft-bearing mice. These anti-tumor functions were associated with enhanced macrophage infiltration and promotion of pro-inflammatory signaling pathways in the tumor microenvironment. Aside from this, IgE is able to promote a pro-inflammatory cytokine profile in monocytic cells and is able to engender ADCC and ADCP of melanoma cells by immune effector cells from patients with advanced melanoma [149]. CSPG4-IgE has been shown to be well tolerated in immunocompetent rodent models [150]. Taken together, these studies point to great potential for future IgE based anti-cancer antigen therapies that may offer new options for the treatment of melanoma by harnessing macrophage effector mechanisms and driving microphages toward pro-inflammatory phenotypes in the tumor microenvironment.

### 7.2. Cell therapies: adapting TAM-based communications in the TME

The underlying principle of Chimeric antigen receptor (CAR) therapy is the modification of immune cells with cell surface chimeric antigen receptors that recognize and bind to specific antigens, on the surface of tumor cells. Adoptive cellular therapy and CAR therapy of T cells, referred to as CAR-T cells, have been successful in the treatment of hematopoietic tumors, but there have also been recent advancements that may lead to applications for the treatment of solid tumors [151]. One approach may be for CAR-T cells to be engineered to target surface markers, such as CD163, CD204 and CD206, predominantly expressed by tumor-promoting TAMs, resulting in their depletion [152]. However, the high plasticity of TAMs and inter- and intra-tumoral heterogeneity, create complexity in the development of efficacious TAM-targeted CAR-T cells. Molecular mapping of macrophage surface markers may offer a chance to profile the distribution of TAMs in the TME and identify novel therapy targets. In addition, the consequences on the immune system of depleting macrophages for a prolonged period are yet unknown. An alternative strategy may therefore be to target and block regulatory macrophage subsets. For example, macrophages expressing FRβ+ are known exert an immunosuppressive effect on T cells, thus FRβ CAR-T cells could be engineered to block the FRβ+ portion of macrophages, reestablishing the cytotoxic properties of T cells [153].

This knowledge has also allowed advancements in engineering of CAR macrophages (CAR-M). TAMs can be constructed with CAR constructs targeting specific cancer antigens, boosting macrophage cytotoxic ability to recognize and phagocytose tumor cells [154]. Alternatively, CAR-M can be reeducated into a pro-inflammatory phenotype, stimulating expression of pro-inflammatory cytokines to induce a tumor-suppressing microenvironment, which subsequently will enhance the immune response of other cells including cytotoxic T cells [155]. To reduce the off-target effects of CAR-M, strategies would need to be adapted to ensure that CAR-M cells are engineered to recognize specific tumor antigens with low and restricted distribution in normal tissues to minimize off-target toxicity and enhance their immune efficacy [154]. CAR-M could also be used in conjunction with established therapies such as immune checkpoint inhibitors [156], overall providing a promising novel therapeutic approach in the treatment of melanoma.

## 8. Concluding thoughts: TAMs in therapy and biomarker development

Here we have discussed the many roles macrophages play, in both the tumorigenesis and treatment of melanoma. TAMs can be continuously influenced by their environment and interact with many other cell types in the TME. This inherent plasticity renders them key players in melanoma and simultaneously an important treatment target. Whilst the TME of melanoma is able to polarize macrophages toward immunoregulatory phenotypes that can support tumor growth, macrophages can also be influenced by exogenous signals, with the potential to be ‘reeducated’ toward more pro-inflammatory subsets.

Licensed therapies for melanoma, including ICIs, MAPKi and T-VEC, can utilize this plasticity for better or worse. For example, anti-CTLA-4 antibodies are reported to enhance macrophage-induced ADCC of Tregs, promoting cancer clearance, whereas MAPKi can promote the secretion of TAM-derived angiogenic factors, supporting tumor growth. Understanding the interactions between macrophages and current therapies, therefore, could be critical for understanding treatment limitations and for improving therapies through multiple mechanisms. Firstly, by enhancing the treatments themselves, for example Fc-engineering to potentiate macrophage-dependent effects of ICI; secondly, priming TAMs, through FcR engagement, CD40 agonism, or TLR stimulation, that can lead to alterations in the TME that will better promote immune responses and enhance the anti-tumor effects of current therapies. And thirdly, developing new cell based therapies, to change the composition of the TME entirely.

Understanding the properties of this most important infiltrating immune cell may be the key to ultimately unlocking better treatments for patients with melanoma, from enhancing of current therapies, to designing new optimized treatments that harness the pro-inflammatory attributes of macrophages to more effectively operate within the immunological constraints of the TME.

## 9. Expert opinion

In this article, we have discussed the multiple roles macrophages play in melanoma, from their ability to promote melanoma cell survival, growth and invasion, angiogenesis and metastasis, to their potential to be key effector cells in current and future antibody treatments and novel cell therapies.

As an archetypal immunogenic tumor, melanoma has been hailed as the success story of immunotherapy, with mAbs that target predominantly the T cell adaptive arm of the immune system having a huge impact on patient outcomes. Despite this success, there remains a substantial proportion of patients who do not benefit from ICIs and/or who suffer toxicities which require withdrawal of treatment. There is a lot of focus on how to potentiate ICI functions, with trials focussing on how best to sequence currently licensed therapies, for example, whether patienst benefit most from ICI followed by MAPKi or vice versa, and which combinations of licensed therapies able to best potentiate therapeutic effects in treatment-resistant patientsfor example, combining radiotherapy and ICIs [157,158].

Immunotherapy has demonstrated that the cancer killing capacity of the immune system can be harnessed and enhanced in the clinical setting. It therefore follows that harnessing the most abundant immune cell in the tumor microenvironment, namely TAMs, could be an effective strategy. TAMs transverse the innate and adaptive immune system and are able to directly trigger killing mechanisms (ADCC/ADCP), as well as secrete pro-inflammatory cytokines that consequently can change the milieu and cellular composition of the TME, including activating T cell and other cellular immune responses.

Targeting TAMs alone has been evaluated, and it is not considered an effective treatment strategy leading to tumor clearance. Furthermore, therapies that have solely focussed on the depletion of macrophages, blocking their recruitment or polarizing these cells, have failed to progress in clinical trials (for example, NCT01494688, NCT00537368, NCT02723006) [148]. Findings from recent pre-clinical studies and clinical trials to-date suggest that TAM-targeted a sufficient as a treatment option and the optimal therapeutic approach has yet to be identified. These failures may be a product of the significant diversity, plasticity and roles of these cells and their ability to be influenced by the tissue microenvironment. This makes targeting one specific function extremely difficult, rendering them a complex and ever evolving target. Pan-macrophage-targeted therapy is associated with systemic toxicity, which presents further challenges. Furthermore, a combination therapeutic approach may help to maximize immunostimulatory activities and minimize associated toxicity.

However, there is an argument that targeting both innate and adaptive immune responses may lead to a synergistic response. Since mAbs have already been shown to be safe and effective treatments in melanoma, designing mAbs with Fc portions that can polarize macrophages or increase their capacity for antibody dependent cell ADCC and ADCP killing mechanisms could be a strategy for improving current treatments. For example, engaging the activatory FcRs on monocytic cells can increase secretion of chemokines which can recruit effector immune cells, and their secretion of cytokines which can activate these cells [149]. If this was coupled with ICIs, which in turn can unleash T cell responses, this could greatly increase the chances of tumor clearance. Aside from this, it is already established that macrophages are able to interact with current ICIs. Understanding this interaction and improving current treatments to make the most of these effector properties of macrophages, for example, modifying Fc-FcR interactions, seems like a clear opportunity to improve currently approved treatments, a process that may be an immediate opportunity when compared to the long process of new drug discovery or costly personalized/autologous cell therapies.

The design of Fc engineered antibodies offers the chance to enhance engagement and signaling through macrophage FcRs. This has been reported as part of targeting CTLA-4 [101] and with novel anti-cancer therapies targeting novel checkpoints [149]. However, creating Fc regions that bind strongly to activatory FcRs promoting phagocytosis by macrophages may lead to exhausted macrophages accumulating in the TME, switching the environment from actively pro-inflammatory to immunoregulatory [159]. The future lies in combinations of therapies and perhaps with sequential treatments, in order to overcome resistance and to ensure that all elements of the immune response are harnessed to achieve cancer cell clearance.

Macrophages are an ever increasingly diverse cell population with a multitude of cellular states and further study characterizing macrophage subsets in melanoma, their functions and how their characteristics and attributes change over time may lead to the discovery of new ways to approach harnessing this abundant tumor infiltrating cell type to treat cancer.

## Article highlights

- Macrophages are a highly diverse and plastic subset of immune cells, with both inflammatory, cancer-killing functions, and immunoregulatory, cancer-promoting functions.
- Melanoma derived factors can influence macrophage polarization to promote pro-tumor functions.
- Macrophages are active players in all current therapies for melanoma: they interact with monoclonal antibody therapy, contribute to MAPKi therapy resistance and play a key role in the mechanism of oncolytic viruses.
- When designing new treatment for melanoma, the interactions with macrophages should be considered. Further to this, specifically targeting macrophages may hold great therapeutic potential.

## References

[1] Data NC. Cancer incidence and mortality. 2022 [cited 2023]. Available from: https://wwwcancerdata.nhs.uk/incidenceandmortality:NHSCancerData

[2] Lin Y, Xu J, Lan H. Tumor-associated macrophages in tumor metastasis: biological roles and clinical therapeutic applications. J Hematol Oncol. 2019;12(1):76. doi: 10.1186/s13045-019-0760-3 31300030 PMC6626377

[3] Sun X, Zhang N, Yin C, et al. Ultraviolet radiation and Melanomagenesis: from mechanism to immunotherapy. Front Oncol. 2020;10:951. doi: 10.3389/fonc.2020.00951 32714859 PMC7343965

[4] Marzagalli M, Ebelt ND, Manuel ER. Unraveling the crosstalk between melanoma and immune cells in the tumor microenvironment. Semin Cancer Biol. 2019;59:236–250. doi: 10.1016/j.semcancer.2019.08.002 31404607

[5] JD W *How Is Immunotherapy For Melanoma Changing The Outlook For Patients?* [cited 2023 Nov 12]. https://www.cancerresearch.org/cancer-types/melanoma2023

[6] Marine JC, Dawson SJ, Dawson MA. Non-genetic mechanisms of therapeutic resistance in cancer. Nat Rev Cancer. 2020;20(12):743–756. doi: 10.1038/s41568-020-00302-4 33033407

[7] Feng Y, Ye Z, Song F, et al. The role of TAMs in tumor microenvironment and new research progress. Stem Cells Int. 2022;2022:5775696. doi: 10.1155/2022/5775696 36004381 PMC9395242

[8] Park MD, Silvin A, Ginhoux F, et al. Macrophages in health and disease. Cell. 2022 Nov 10;185(23):4259–4279. doi: 10.1016/j.cell.2022.10.007 36368305 PMC9908006

[9] Hoeffel G, Chen J, Lavin Y, et al. C-Myb+ erythro-myeloid progenitor-derived fetal monocytes give rise to adult tissue-resident macrophages. Immunity. 2015 Apr 21;42(4):665–678. doi: 10.1016/j.immuni.2015.03.011 25902481 PMC4545768

[10] Laviron M, Boissonnas A. Ontogeny of tumor-associated macrophages. Front Immunol. 2019;10:1799. doi: 10.3389/fimmu.2019.01799 31417566 PMC6684758

[11] Christofides A, Strauss L, Yeo A, et al. The complex role of tumor-infiltrating macrophages. Nat Immunol. 2022;23(8):1148–1156. 35879449 10.1038/s41590-022-01267-2 PMC10754321

[12] Lahmar Q, Keirsse J, Laoui D, et al. Tissue-resident versus monocyte-derived macrophages in the tumor microenvironment. Biochim Biophys Acta. 2016;1865(1):23–34. 26145884 10.1016/j.bbcan.2015.06.009

[13] Franklin RA, Li MO. Ontogeny of tumor-associated macrophages and its implication in cancer regulation. Trends Cancer. 2016 1;2(1):20–34. doi: 10.1016/j.trecan.2015.11.004 26949745 PMC4772875

[14] Hirayama D, Iida T, Nakase H. The phagocytic function of macrophage-enforcing innate immunity and tissue homeostasis. Int J Mol Sci. 2017;19(1):92. doi: 10.3390/ijms19010092 29286292 PMC5796042

[15] Muntjewerff EM, Meesters LD, van den Bogaart G. Antigen cross-presentation by macrophages. Front Immunol. 2020;11:1276. doi: 10.3389/fimmu.2020.01276 32733446 PMC7360722

[16] Shapouri-Moghaddam A, Mohammadian S, Vazini H, et al. Macrophage plasticity, polarization, and function in health and disease. J Cell Physiol. 2018;233(9):6425–6440. 10.1002/jcp.26429 PMC13482560 29319160

[17] Murray PJ. Macrophage Polarization. Annu Rev Physiol. 2017 10;79(1):541–566. doi: 10.1146/annurev-physiol-022516-034339 27813830

[18] Akhter N, Wilson A, Thomas R, et al. ROS/TNF-α crosstalk triggers the expression of IL-8 and MCP-1 in human monocytic THP-1 cells via the NF-κB and ERK1/2 mediated signaling. Int J Mol Sci. 2021;22(19):10519. doi: 10.3390/ijms221910519 34638857 PMC8508672

[19] Marconi C, Bianchini F, Mannini A, et al. Tumoral and macrophage uPAR and MMP-9 contribute to the invasiveness of B16 murine melanoma cells. Clin Exp Metastasis. 2008;25(3):225–31. doi: 10.1007/s10585-007-9136-0 18071911

[20] Rőszer T. Understanding the mysterious M2 macrophage through activation markers and effector mechanisms. Mediators Inflamm. 2015;2015:1–16. doi: 10.1155/2015/816460 PMC4452191 26089604

[21] Falleni M, Savi F, Tosi D, et al. M1 and M2 macrophages’ clinicopathological significance in cutaneous melanoma. Melanoma Res. 2017 Jun;27(3):200–210. 28272106 10.1097/CMR.0000000000000352

[22] Ma RY, Black A, Qian BZ. Macrophage diversity in cancer revisited in the era of single-cell omics. Trends Immunol. 2022;43(7):546–563. doi: 10.1016/j.it.2022.04.008. 35690521

[23] Scali E, Mignogna C, Di Vito A, et al. Inflammation and macrophage polarization in cutaneous melanoma: Histopathological and immunohistochemical study. Int J Immunopathol Pharmacol. 2016;29(4):715–719. 27387897 10.1177/0394632016650895 PMC5806828

[24] Mashima H, Zhang R, Kobayashi T, et al. Generation of GM-CSF-producing antigen-presenting cells that induce a cytotoxic T cell-mediated antitumor response. Oncoimmunology. 2020 Sep 6;9(1):1814620. doi: 10.1080/2162402X.2020.1814620 33457097 PMC7781730

[25] Bardi GT, Smith MA, Hood JL. Melanoma exosomes promote mixed M1 and M2 macrophage polarization. Cytokine. 2018 May;105:63–72. doi: 10.1016/j.cyto.2018.02.002 29459345 PMC5857255

[26] Huber R, Meier B, Otsuka A, et al. Tumour hypoxia promotes melanoma growth and metastasis via high mobility group box-1 and M2-like macrophages. Sci Rep. 2016;6(1):29914. doi: 10.1038/srep29914 27426915 PMC4947927

[27] Tan Y, Wang M, Zhang Y, et al. Tumor-associated macrophages: a potential target for cancer therapy. Front Oncol. 2021;11:693517. doi: 10.3389/fonc.2021.693517 34178692 PMC8222665

[28] Tong Y, Guo YJ, Zhang Q, et al. Combined treatment with dihydrotestosterone and lipopolysaccharide modulates prostate homeostasis by upregulating TNF-α from M1 macrophages and promotes proliferation of prostate stromal cells. Asian J Androl. 2022;24(5):513–520. doi: 10.4103/aja2021114 34975070 PMC9491040

[29] Lim TS, Goh JK, Mortellaro A, et al. CD80 and CD86 differentially regulate mechanical interactions of T-cells with antigen-presenting dendritic cells and B-cells. PloS One. 2012;7(9):e45185. doi: 10.1371/journal.pone.0045185 23024807 PMC3443229

[30] Bi S, Huang W, Chen S, et al. Cordyceps militaris polysaccharide converts immunosuppressive macrophages into M1-like phenotype and activates T lymphocytes by inhibiting the PD-L1/PD-1 axis between TAMs and T lymphocytes. Int j biol macromol. 2020;150:261–280. doi: 10.1016/j.ijbiomac.2020.02.050 32044366

[31] Liu C, Chikina M, Deshpande R, et al. Treg cells promote the SREBP1-dependent metabolic fitness of tumor-promoting macrophages via repression of CD8. Immunity. 2019 Aug 20;51(2):381–397.e6. doi: 10.1016/j.immuni.2019.06.017 31350177 PMC6703933

[32] Ni R, Jiang L, Zhang C, et al. Biologic mechanisms of macrophage phenotypes responding to infection and the novel therapies to moderate inflammation. Int J Mol Sci. 2023;24(9):8358. doi: 10.3390/ijms24098358 37176064 PMC10179618

[33] Liu PS, Chen YT, Li X, et al. CD40 signal rewires fatty acid and glutamine metabolism for stimulating macrophage anti-tumorigenic functions. Nat Immunol. 2023;24(3):452–462. 36823405 10.1038/s41590-023-01430-3 PMC9977680

[34] Kashyap AS, Schmittnaegel M, Rigamonti N, et al. Optimized antiangiogenic reprogramming of the tumor microenvironment potentiates CD40 immunotherapy. Proc Natl Acad Sci USA. 2020 Jan 7;117(1):541–551. doi: 10.1073/pnas.1902145116 31889004 PMC6955310

[35] Long KB, Gladney WL, Tooker GM, et al. IFNγ and CCL2 cooperate to redirect tumor-infiltrating monocytes to degrade fibrosis and enhance chemotherapy efficacy in pancreatic carcinoma. Cancer Discov. 2016;6(4):400–413. 26896096 10.1158/2159-8290.CD-15-1032 PMC4843521

[36] Xing Q, Feng Y, Sun H, et al. Scavenger receptor MARCO contributes to macrophage phagocytosis and clearance of tumor cells. Exp Cell Res. 2021;408(2):112862. doi: 10.1016/j.yexcr.2021.112862 34626585

[37] Gu C, Wiest M, Zhang W, et al. Cancer cells promote immune regulatory function of macrophages by upregulating scavenger receptor MARCO expression. J Immunol. 2023 Jul 1;211(1):57–70. doi: 10.4049/jimmunol.2300029 37212598

[38] La Fleur L, Botling J, He F, et al. Targeting MARCO and IL37R on immunosuppressive macrophages in lung cancer blocks regulatory t cells and supports cytotoxic lymphocyte function. Cancer Res. 2021;81(4):956–967. doi: 10.1158/0008-5472.CAN-20-1885. 33293426

[39] Eisinger S, Sarhan D, Boura VF, et al. Targeting a scavenger receptor on tumor-associated macrophages activates tumor cell killing by natural killer cells. Proc Natl Acad Sci USA. 202015;117(50):32005–32016. doi: 10.1073/pnas.2015343117 33229588 PMC7750482

[40] Ohkuri T, Kosaka A, Nagato T, et al. Effects of STING stimulation on macrophages: STING agonists polarize into “classically” or “alternatively” activated macrophages? Hum Vaccin Immunother. 2018;14(2):285–287. doi: 10.1080/21645515.2017.1395995 29064738 PMC5806643

[41] Sato S, Sawada Y, Nakamura M. STING signaling and skin cancers. Cancers (Basel). 2021 Nov 9;13(22):5603. doi: 10.3390/cancers13225603 34830754 PMC8615888

[42] Sun Y, Hu H, Liu Z, et al. Macrophage STING signaling promotes NK cell to suppress colorectal cancer liver metastasis via 4-1BBL/4-1BB co-stimulation. J Immunother Cancer. 2023;11(3):e006481. 36927529 10.1136/jitc-2022-006481 PMC10030919

[43] Zhang Y, Zhai Q, Feng X, et al. Cancer cell-intrinsic STING is associated with CD8 + T-cell infiltration and might serve as a potential immunotherapeutic target in hepatocellular carcinoma. Clin Transl Oncol. 2021;23(7):1314–1324. 33502741 10.1007/s12094-020-02519-z

[44] Wu YT, Fang Y, Wei Q, et al. Tumor-targeted delivery of a STING agonist improvescancer immunotherapy. Proc Natl Acad Sci USA. 2022 Dec;119(49):e2214278119. doi: 10.1073/pnas.2214278119 36442099 PMC9894229

[45] Kobayashi T, Oishi K, Okamura A, et al. Regulatory B1a cells suppress melanoma tumor immunity via IL-10 production and Inhibiting T Helper Type 1 cytokine production in tumor-infiltrating CD8+ T cells. J Invest Dermatol. 2019;139(7):1535–1544.e1. 30836062 10.1016/j.jid.2019.02.016

[46] Harris RJ, Willsmore Z, Laddach R, et al. Enriched circulating and tumor-resident TGF-β + regulatory B cells in patients with melanoma promote FOXP3 + tregs. Oncoimmunology. 2022;11(1):2104426. doi: 10.1080/2162402X.2022.2104426 35909944 PMC9336482

[47] Laurindo MF, Thies FG, Perez EC, et al. B16 melanoma cells increase B-1 cell survival, IL-10 production and radioresistance in vitro. Immunobiology. 2013 Apr;218(4):609–619. 22954710 10.1016/j.imbio.2012.07.032

[48] Fridman WH, Meylan M, Petitprez F, et al. B cells and tertiary lymphoid structures as determinants of tumour immune contexture and clinical outcome. Nat Rev Clin Oncol. 2022;19(7):441–457. 35365796 10.1038/s41571-022-00619-z

[49] Zhang B, Vogelzang A, Miyajima M, et al. B cell-derived GABA elicits IL-10. Nature. 2021;599(7885):471–476. 34732892 10.1038/s41586-021-04082-1 PMC8599023

[50] Chen C, Park B, Ragonnaud E, et al. Cancer co-opts differentiation of B-cell precursors into macrophage-like cells. Nat Commun. 2022;13(1):5376. doi: 10.1038/s41467-022-33117-y 36104343 PMC9474882

[51] Cheng X, Wang H, Wang Z, et al. Tumor-associated myeloid cells in cancer immunotherapy. J Hematol Oncol. 2023;16(1):71. doi: 10.1186/s13045-023-01473-x 37415162 PMC10324139

[52] Kersten K, Coffelt SB, Hoogstraat M, et al. Mammary tumor-derived CCL2 enhances pro-metastatic systemic inflammation through upregulation of IL1β in tumor-associated macrophages. Oncoimmunology. 2017;6(8):e1334744. doi: 10.1080/2162402X.2017.1334744 28919995 PMC5593698

[53] Ferrucci PF, Ascierto PA, Pigozzo J, et al. Baseline neutrophils and derived neutrophil-to-lymphocyte ratio: prognostic relevance in metastatic melanoma patients receiving ipilimumab. Ann Oncol. 2016;27(4):732–8. 26802161 10.1093/annonc/mdw016

[54] Anselmi M, Fontana F, Marzagalli M, et al. Melanoma stem cells educate neutrophils to support cancer progression. Cancers (Basel). 2022 Jul 13;14(14):3391. doi: 10.3390/cancers14143391 35884452 PMC9317939

[55] Bruger AM, Dorhoi A, Esendagli G, et al. How to measure the immunosuppressive activity of MDSC: assays, problems and potential solutions. Cancer Immunol Immunother. 2019 Apr;68(4):631–644. 29785656 10.1007/s00262-018-2170-8 PMC11028070

[56] See P, Dutertre CA, Chen J. et al. Mapping the human DC lineage through the integration of high-dimensional techniques. Science. 2017 Jun 9;356(6342). doi: 10.1126/science.aag3009 PMC7611082 28473638

[57] Grabowska J, Lopez-Venegas MA, Affandi AJ, et al. CD169. Front Immunol. 2018;9:2472. doi: 10.3389/fimmu.2018.02472 30416504 PMC6212557

[58] Kvedaraite E, Ginhoux F. Human dendritic cells in cancer. Sci Immunol. 2022 Apr;7(70):eabm9409. doi: 10.1126/sciimmunol.abm9409 35363544

[59] Di Blasio S, van Wigcheren GF, Becker A, et al. The tumour microenvironment shapes dendritic cell plasticity in a human organotypic melanoma culture. Nat Commun. 2020 Jun 2;11(1):2749. doi: 10.1038/s41467-020-16583-0 32488012 PMC7265463

[60] Gabrilovich DI, Nagaraj S. Myeloid-derived suppressor cells as regulators of the immune system. Nat Rev Immunol. 2009 Mar;9(3):162–74. doi: 10.1038/nri2506 19197294 PMC2828349

[61] Hourani T, Holden JA, Li W, et al. Tumor associated macrophages: origin, recruitment, phenotypic diversity, and targeting. Front Oncol. 2021;11:788365. doi: 10.3389/fonc.2021.788365 34988021 PMC8722774

[62] Zhou J, Tang Z, Gao S, et al. Tumor-associated macrophages: recent insights and therapies. Front Oncol. 2020;10:188. doi: 10.3389/fonc.2020.00188 32161718 PMC7052362

[63] Gordon SR, Maute RL, Dulken BW, et al. PD-1 expression by tumour-associated macrophages inhibits phagocytosis and tumour immunity. Nature. 2017 May 25;545(7655):495–499. doi: 10.1038/nature22396 28514441 PMC5931375

[64] Mantovani A, Allavena P, Marchesi F, et al. Macrophages as tools and targets in cancer therapy. Nat Rev Drug Discov. 2022 Nov;21(11):799–820. 35974096 10.1038/s41573-022-00520-5 PMC9380983

[65] Martin P, Gurevich DB. Macrophage regulation of angiogenesis in health and disease. Semin Cell Dev Biol. 2021 Nov;119:101–110. doi: 10.1016/j.semcdb.2021.06.010 34330619

[66] Kumar R, Mickael C, Kassa B, et al. Interstitial macrophage-derived thrombospondin-1 contributes to hypoxia-induced pulmonary hypertension. Cardiovasc Res. 2020 Oct 1;116(12):2021–2030. doi: 10.1093/cvr/cvz304 31710666 PMC7519884

[67] Hwang I, Kim JW, Ylaya K, et al. Tumor-associated macrophage, angiogenesis and lymphangiogenesis markers predict prognosis of non-small cell lung cancer patients. J Transl Med. 2020 Nov 23;18(1):443. doi: 10.1186/s12967-020-02618-z 33228719 PMC7686699

[68] Torisu H, Ono M, Kiryu H, et al. Macrophage infiltration correlates with tumor stage and angiogenesis in human malignant melanoma: possible involvement of TNFα and IL-1α. Int J Cancer. 2000 Jan 15;85(2):182–188. doi: 10.1002/(SICI)1097-0215(20000115)85:2<182:AID-IJC6>3.0.CO;2-M 10629075

[69] Domokos A, Varga Z, Jambrovics K, et al. The transcriptional control of the VEGFA-VEGFR1 (FLT1) axis in alternatively polarized murine and human macrophages. Front Immunol. 2023;14:1168635. doi: 10.3389/fimmu.2023.1168635 37215144 PMC10192733

[70] Benyahia Z, Gaudy-Marqueste C, Berenguer-Daizé C, et al. Adrenomedullin secreted by melanoma cells promotes melanoma tumor growth through angiogenesis and Lymphangiogenesis. Cancers (Basel). 2022 Nov 29;14(23):5909. doi: 10.3390/cancers14235909 36497391 PMC9738606

[71] Lee J, Jin YJ, Lee MS, et al. Macrophage inhibitory cytokine-1 produced by melanoma cells contributes to melanoma tumor growth and metastasis in vivo by enhancing tumor vascularization. Melanoma Res. 2022 Feb 1;32(1):1–10. doi: 10.1097/CMR.0000000000000790 34939980

[72] Muniyan S, Pothuraju R, Seshacharyulu P, et al. Macrophage inhibitory cytokine-1 in cancer: beyond the cellular phenotype. Cancer Lett. 2022 Jun 28;536:215664. doi: 10.1016/j.canlet.2022.215664 35351601 PMC9088220

[73] Roche J. The epithelial-to-mesenchymal transition in cancer. Cancers (Basel). 2018 Feb 16;10(2):52. doi: 10.3390/cancers10020052 29462906 PMC5836084

[74] Fu XT, Dai Z, Song K, et al. Macrophage-secreted IL-8 induces epithelial-mesenchymal transition in hepatocellular carcinoma cells by activating the JAK2/STAT3/Snail pathway. Int J Oncol. 2015 Feb;46(2):587–96. 25405790 10.3892/ijo.2014.2761

[75] Ravi J, Elbaz M, Wani NA, et al. Cannabinoid receptor-2 agonist inhibits macrophage induced EMT in non-small cell lung cancer by downregulation of EGFR pathway. Mol Carcinog. 2016 Dec;55(12):2063–2076. 26741322 10.1002/mc.22451 PMC7063844

[76] Kawata M, Koinuma D, Ogami T, et al. TGF-β-induced epithelial-mesenchymal transition of A549 lung adenocarcinoma cells is enhanced by pro-inflammatory cytokines derived from RAW 264.7 macrophage cells. J Biochem. 2012 Feb;151(2):205–16. 22161143 10.1093/jb/mvr136

[77] Delprat V, Michiels C. A bi-directional dialog between vascular cells and monocytes/macrophages regulates tumor progression. Cancer Metastasis Rev. 2021 Jun;40(2):477–500. doi: 10.1007/s10555-021-09958-2 33783686 PMC8213675

[78] Sevenich L, Joyce JA. Pericellular proteolysis in cancer. Genes Dev. 2014 Nov 1;28(21):2331–47. doi: 10.1101/gad.250647.114 25367033 PMC4215179

[79] Onal S, Turker-Burhan M, Bati-Ayaz G, et al. Breast cancer cells and macrophages in a paracrine-juxtacrine loop. Biomaterials. 2021 Jan;267:120412. doi: 10.1016/j.biomaterials.2020.120412 33161320

[80] Cantelli G, Orgaz JL, Rodriguez-Hernandez I, et al. TGF-β-induced transcription sustains amoeboid melanoma migration and dissemination. Curr Biol. 2015 Nov 16;25(22):2899–914. doi: 10.1016/j.cub.2015.09.054 26526369 PMC4651903

[81] Sanz-Moreno V, Gaggioli C, Yeo M, et al. ROCK and JAK1 signaling cooperate to control actomyosin contractility in tumor cells and stroma. Cancer Cell. 2011 Aug 16;20(2):229–45. doi: 10.1016/j.ccr.2011.06.018 21840487

[82] Georgouli M, Herraiz C, Crosas-Molist E, et al. Regional activation of myosin II in cancer cells drives tumor progression via a secretory cross-talk with the immune microenvironment. Cell. 2019 Feb 7;176(4):757–774.e23. doi: 10.1016/j.cell.2018.12.038 30712866 PMC6370915

[83] Wong PP, Muñoz-Félix JM, Hijazi M, et al. Cancer burden is controlled by mural cell-β3-integrin regulated crosstalk with tumor cells. Cell. 2020 Jun 11;181(6):1346–1363.e21. doi: 10.1016/j.cell.2020.02.003 32473126

[84] Orgaz JL, Crosas-Molist E, Sadok A, et al. Myosin II reactivation and cytoskeletal remodeling as a hallmark and a vulnerability in melanoma therapy resistance. Cancer Cell. 2020 Jan 13;37(1):85–103.e9. doi: 10.1016/j.ccell.2019.12.003 31935375 PMC6958528

[85] Qian B, Deng Y, Im JH, et al. A distinct macrophage population mediates metastatic breast cancer cell extravasation, establishment and growth. PloS One. 2009 Aug 10;4(8):e6562. doi: 10.1371/journal.pone.0006562 19668347 PMC2721818

[86] Martinek J, Lin J, Kim KI, et al. Transcriptional profiling of macrophages in situ in metastatic melanoma reveals localization-dependent phenotypes and function. Cell Rep Med. 2022 May 17;3(5):100621. doi: 10.1016/j.xcrm.2022.100621 35584631 PMC9133468

[87] Holt MP, Punkosdy GA, Glass DD, et al. TCR signaling and CD28/CTLA-4 signaling cooperatively modulate T regulatory cell homeostasis. J Immunol. 2017 Feb 15;198(4):1503–1511. doi: 10.4049/jimmunol.1601670 28053234 PMC5296272

[88] Garcia-Diaz A, Shin DS, Moreno BH, et al. Interferon receptor signaling pathways regulating PD-L1 and PD-L2 expression. Cell Rep. 2017 May 9;19(6):1189–1201. doi: 10.1016/j.celrep.2017.04.031 28494868 PMC6420824

[89] Zhao Y, Harrison DL, Song Y, et al. Antigen-Presenting Cell-Intrinsic PD-1 Neutralizes PD-L1 in cis to Attenuate PD-1 Signaling in T Cells. Cell Rep. 2018 Jul 10;24(2):379–390.e6. doi: 10.1016/j.celrep.2018.06.054 29996099 PMC6093302

[90] Willsmore ZN, Coumbe BGT, Crescioli S, et al. Combined anti-PD-1 and anti-CTLA-4 checkpoint blockade: treatment of melanoma and immune mechanisms of action. Eur J Immunol. 2021 Mar;51(3):544–556. 33450785 10.1002/eji.202048747

[91] Hamid O, Robert C, Daud A, et al. Five-year survival outcomes for patients with advanced melanoma treated with pembrolizumab in KEYNOTE-001. Ann Oncol. 2019 Apr 1;30(4):582–588. doi: 10.1093/annonc/mdz011 30715153 PMC6503622

[92] Hodi FS, Chiarion-Sileni V, Gonzalez R, et al. Nivolumab plus ipilimumab or nivolumab alone versus ipilimumab alone in advanced melanoma (CheckMate 067): 4-year outcomes of a multicentre, randomised, phase 3 trial. Lancet Oncol. 2018 Nov;19(11):1480–1492. 30361170 10.1016/S1470-2045(18)30700-9

[93] Larkin J, Chiarion-Sileni V, Gonzalez R, et al. Five-Year Survival with Combined Nivolumab and Ipilimumab in Advanced Melanoma. N Engl J Med. 2019 Oct 17;381(16):1535–1546. doi: 10.1056/NEJMoa1910836 31562797

[94] Yu J, Song Y, Tian W. How to select IgG subclasses in developing anti-tumor therapeutic antibodies. J Hematol Oncol. 2020 May 5;13(1):45. doi: 10.1186/s13045-020-00876-4 32370812 PMC7201658

[95] Chenoweth AM, Wines BD, Anania JC, et al. Harnessing the immune system via FcγR function in immune therapy: a pathway to next-gen mAbs. Immunol Cell Biol. 2020 Apr;98(4):287–304. doi: 10.1111/imcb.12326 32157732 PMC7228307

[96] Bournazos S, Gupta A, Ravetch JV. The role of IgG Fc receptors in antibody-dependent enhancement. Nat Rev Immunol. 2020 Oct;20(10):633–643. doi: 10.1038/s41577-020-00410-0 32782358 PMC7418887

[97] Crescioli S, Correa I, Karagiannis P, et al. IgG4 characteristics and functions in cancer immunity. Curr Allergy Asthma Rep. 2016 Jan;16(1):7. 26742760 10.1007/s11882-015-0580-7 PMC4705142

[98] Karagiannis P, Gilbert AE, Josephs DH, et al. IgG4 subclass antibodies impair antitumor immunity in melanoma. J Clin Invest. 2013 Apr;123(4):1457–74. 23454746 10.1172/JCI65579 PMC3613918

[99] Arlauckas SP, Garris CS, Kohler RH, et al. In vivo imaging reveals a tumor-associated macrophage-mediated resistance pathway in anti-PD-1 therapy. Sci Transl Med. 2017 May 10;9(389). doi: 10.1126/scitranslmed.aal3604 PMC5734617 28490665

[100] Zhang T, Song X, Xu L, et al. The binding of an anti-PD-1 antibody to FcγRΙ has a profound impact on its biological functions. Cancer Immunol Immunother. 2018 Jul;67(7):1079–1090. 29687231 10.1007/s00262-018-2160-x PMC6006217

[101] Romano E, Kusio-Kobialka M, Foukas PG, et al. Ipilimumab-dependent cell-mediated cytotoxicity of regulatory T cells ex vivo by nonclassical monocytes in melanoma patients. Proc Natl Acad Sci USA. 2015 May 12;112(19):6140–5. doi: 10.1073/pnas.1417320112 25918390 PMC4434760

[102] Simpson TR, Li F, Montalvo-Ortiz W, et al. Fc-dependent depletion of tumor-infiltrating regulatory T cells co-defines the efficacy of anti–CTLA-4 therapy against melanoma. J Exp Med. 2013 Aug 26;210(9):1695–1710. doi: 10.1084/jem.20130579 23897981 PMC3754863

[103] Yofe I, Landsberger T, Yalin A, et al. Anti-CTLA-4 antibodies drive myeloid activation and reprogram the tumor microenvironment through FcγR engagement and type I interferon signaling. Nat Cancer. 2022 Nov;3(11):1336–1350. doi: 10.1038/s43018-022-00447-1 36302895

[104] Hartley GP, Chow L, Ammons DT, et al. Programmed cell death ligand 1 (PD-L1) signaling regulates macrophage proliferation and activation. Cancer Immunol Res. 2018 Oct;6(10):1260–1273. 30012633 10.1158/2326-6066.CIR-17-0537

[105] Tang H, Liang Y, Anders RA, et al. PD-L1 on host cells is essential for PD-L1 blockade–mediated tumor regression. J Clin Invest. 2018 Feb 1;128(2):580–588. doi: 10.1172/JCI96061 29337303 PMC5785245

[106] Lin W, Xu D, Austin CD, et al. Function of CSF1 and IL34 in macrophage homeostasis, inflammation, and cancer. Front Immunol. 2019;10:2019. doi: 10.3389/fimmu.2019.02019 31552020 PMC6736990

[107] Zhu Y, Knolhoff BL, Meyer MA, et al. CSF1/CSF1R blockade reprograms tumor-infiltrating macrophages and improves response to T-cell checkpoint immunotherapy in pancreatic cancer models. Cancer Res. 2014 Sep 15;74(18):5057–69. doi: 10.1158/0008-5472.CAN-13-3723 25082815 PMC4182950

[108] Cassetta L, Pollard JW. Targeting macrophages: therapeutic approaches in cancer. Nat Rev Drug Discov. 2018 Dec;17(12):887–904. doi: 10.1038/nrd.2018.169 30361552

[109] Gomez-Roca C, Cassier P, Zamarin D, et al. Anti-CSF-1R emactuzumab in combination with anti-PD-L1 atezolizumab in advanced solid tumor patients naïve or experienced for immune checkpoint blockade. J Immunother Cancer. 2022 May;10(5):e004076. 35577503 10.1136/jitc-2021-004076 PMC9114963

[110] Gomez-Roca CA, Italiano A, Le Tourneau C, et al. Phase I study of emactuzumab single agent or in combination with paclitaxel in patients with advanced/metastatic solid tumors reveals depletion of immunosuppressive M2-like macrophages. Ann Oncol. 2019 Aug 1;30(8):1381–1392. doi: 10.1093/annonc/mdz163 31114846 PMC8887589

[111] Lu D, Ni Z, Liu X, et al. Beyond T cells: understanding the role of PD-1/PD-L1 in tumor-associated macrophages. J Immunol Res. 2019;2019:1919082. doi: 10.1155/2019/1919082 31781673 PMC6875348

[112] Xiong H, Mittman S, Rodriguez R, et al. Anti-PD-L1 treatment results in functional remodeling of the macrophage compartment. Cancer Res. 2019 Apr 1;79(7):1493–1506. doi: 10.1158/0008-5472.CAN-18-3208 30679180

[113] Fang WB, Yao M, Brummer G, et al. Targeted gene silencing of CCL2 inhibits triple negative breast cancer progression by blocking cancer stem cell renewal and M2 macrophage recruitment. Oncotarget. 2016 Aug 2;7(31):49349–49367. doi: 10.18632/oncotarget.9885 27283985 PMC5226513

[114] Peranzoni E, Lemoine J, Vimeux L, et al. Macrophages impede CD8 T cells from reaching tumor cells and limit the efficacy of anti-PD-1 treatment. Proc Natl Acad Sci USA. 2018 Apr 24;115(17):E4041–E4050. doi: 10.1073/pnas.1720948115 29632196 PMC5924916

[115] Xu M, Wang Y, Xia R, et al. Role of the CCL2-CCR2 signalling axis in cancer: mechanisms and therapeutic targeting. Cell Prolif. 2021 Oct;54(10):e13115. 34464477 10.1111/cpr.13115 PMC8488570

[116] Tokunaga R, Zhang W, Naseem M, et al. CXCL9, CXCL10, CXCL11/CXCR3 axis for immune activation–a target for novel cancer therapy. Cancer Treat Rev. 2018 Feb;63:40–47. doi: 10.1016/j.ctrv.2017.11.007 29207310 PMC5801162

[117] House IG, Savas P, Lai J, et al. Macrophage-derived CXCL9 and CXCL10 are required for antitumor immune responses following immune checkpoint blockade. Clin Cancer Res. 2020 Jan 15;26(2):487–504. doi: 10.1158/1078-0432.CCR-19-1868 31636098

[118] Batlle E, Massagué J. Transforming growth factor-β signaling in immunity and cancer. Immunity. 2019 Apr 16;50(4):924–940. doi: 10.1016/j.immuni.2019.03.024 30995507 PMC7507121

[119] Konkel JE, Zhang D, Zanvit P, et al. Transforming growth factor-β signaling in regulatory T cells controls T helper-17 cells and tissue-specific immune responses. Immunity. 2017 Apr 18;46(4):660–674. doi: 10.1016/j.immuni.2017.03.015 28423340 PMC12230991

[120] Lim YW, Coles GL, Sandhu SK, et al. Single-cell transcriptomics reveals the effect of PD-L1/TGF-β blockade on the tumor microenvironment. BMC Biol. 2021 May 25;19(1):107. doi: 10.1186/s12915-021-01034-z 34030676 PMC8147417

[121] Yu J, Green MD, Li S, et al. Liver metastasis restrains immunotherapy efficacy via macrophage-mediated T cell elimination. Nat Med. 2021 Jan;27(1):152–164. 33398162 10.1038/s41591-020-1131-x PMC8095049

[122] Weiss SA, Djureinovic D, Jessel S, et al. A phase I study of APX005M and cabiralizumab with or without nivolumab in patients with melanoma, kidney cancer, or non-small cell lung cancer resistant to anti-PD-1/PD-L1. Clin Cancer Res. 2021 Sep 1;27(17):4757–4767. doi: 10.1158/1078-0432.CCR-21-0903 34140403 PMC9236708

[123] Huffaker TB, Lee SH, Tang WW, et al. Antitumor immunity is defective in T cell–specific microRNA-155–deficient mice and is rescued by immune checkpoint blockade. J Biol Chem. 2017 Nov 10;292(45):18530–18541. doi: 10.1074/jbc.M117.808121 28912267 PMC5682963

[124] Network CGA, Akdemir K, Aksoy B. Genomic classification of cutaneous melanoma. Cell. 2015 Jun 18;161(7):1681–1696. doi: 10.1016/j.cell.2015.05.044 26091043 PMC4580370

[125] Alqathama A. BRAF in malignant melanoma progression and metastasis: potentials and challenges. Am J Cancer Res. 2020;10(4):1103–1114. 32368388 PMC7191094

[126] Robert C, Grob JJ, Stroyakovskiy D, et al. Five-year outcomes with Dabrafenib plus trametinib in metastatic melanoma. N Engl J Med. 2019 Aug 15;381(7):626–636. doi: 10.1056/NEJMoa1904059 31166680

[127] Kim D, An L, Moon J, et al. Ccr2+ monocyte-derived macrophages influence trajectories of acquired therapy resistance in braf-mutant melanoma. Cancer Res. 2023 Jul 14;83(14):2328–2344. doi: 10.1158/0008-5472.CAN-22-2841 37195124 PMC10478295

[128] Mikubo M, Inoue Y, Liu G, et al. Mechanism of drug tolerant persister cancer cells: the landscape and clinical implication for therapy. J Thorac Oncol. 2021 Nov;16(11):1798–1809. 34352380 10.1016/j.jtho.2021.07.017

[129] Zhang Z, Tan Y, Huang C, et al. Redox signaling in drug-tolerant persister cells as an emerging therapeutic target. EBioMedicine. 2023 Mar;89:104483. doi: 10.1016/j.ebiom.2023.104483 36827719 PMC9982619

[130] Giricz O, Mo Y, Dahlman KB. et al. The RUNX1/IL-34/CSF-1R axis is an autocrinally regulated modulator of resistance to BRAF-V600E inhibition in melanoma. JCI Insight. 2018 Jul 26;3(14). doi: 10.1172/jci.insight.120422 PMC6124424 30046005

[131] Atzori MG, Ceci C, Ruffini F, et al. Role of VEGFR-1 in melanoma acquired resistance to the BRAF inhibitor vemurafenib. J Cell Mol Med. 2020 Jan;24(1):465–475. 31758648 10.1111/jcmm.14755 PMC6933379

[132] Koizumi K, Shintani T, Hayashido Y, et al. VEGF-A promotes the motility of human melanoma cells through the VEGFR1-PI3K/Akt signaling pathway. Vitro Cell Dev Biol Anim. 2022 Sep;58(8):758–770. 10.1007/s11626-022-00717-3 PMC9550759 35997849

[133] Fattore L, Cafaro G, Di Martile M, et al. Oncosuppressive miRnas loaded in lipid nanoparticles potentiate targeted therapies in BRAF-mutant melanoma by inhibiting core escape pathways of resistance. Oncogene. 2023 Jan;42(4):293–307. 36418472 10.1038/s41388-022-02547-9 PMC9684877

[134] Alexander ET, El Naggar O, Fahey E, et al. Harnessing the polyamine transport system to treat BRAF inhibitor-resistant melanoma. Cancer Biol Ther. 2021 Mar 4;22(3):225–237. doi: 10.1080/15384047.2021.1883185 33602034 PMC8043175

[135] Andtbacka RH, Agarwala SS, Ollila DW, et al. Cutaneous head and neck melanoma in OPTiM, a randomized phase 3 trial of talimogene laherparepvec versus granulocyte-macrophage colony-stimulating factor for the treatment of unresected stage IIIB/IIIC/IV melanoma. Head Neck. 2016 Dec;38(12):1752–1758. 27407058 10.1002/hed.24522 PMC5129499

[136] Andtbacka RHI, Collichio F, Harrington KJ, et al. Final analyses of OPTiM: a randomized phase III trial of talimogene laherparepvec versus granulocyte-macrophage colony-stimulating factor in unresectable stage III-IV melanoma. J Immunother Cancer. 2019 Jun 6;7(1):145. doi: 10.1186/s40425-019-0623-z 31171039 PMC6554874

[137] Ghasemi M, Abbasi L, Ghanbari Naeini L, et al. Dendritic cells and natural killer cells: the road to a successful oncolytic virotherapy. Front Immunol. 2022;13:950079. doi: 10.3389/fimmu.2022.950079 36703982 PMC9871831

[138] Jennings VA, Scott GB, Rose AMS, et al. Potentiating oncolytic virus-induced immune-mediated tumor cell killing using histone deacetylase inhibition. Mol Ther. 2019 Jun 5;27(6):1139–1152. doi: 10.1016/j.ymthe.2019.04.008 31053413 PMC6554638

[139] Berkeley RA, Steele LP, Mulder AA, et al. Antibody-neutralized reovirus is effective in oncolytic virotherapy. Cancer Immunol Res. 2018 Oct;6(10):1161–1173. 30209061 10.1158/2326-6066.CIR-18-0309

[140] Liikanen I, Basnet S, Quixabeira DCA, et al. Oncolytic adenovirus decreases the proportion of TIM-3+subset of tumor-infiltrating CD8+T cells with correlation to improved survival in patients with cancer. J Immunother Cancer. 2022 Feb;10(2):e003490. 35193929 10.1136/jitc-2021-003490 PMC8867324

[141] Vidyarthi A, Khan N, Agnihotri T, et al. TLR-3 stimulation skews M2 macrophages to M1 through IFN-αβ signaling and restricts tumor progression. Front Immunol. 2018;9:1650. doi: 10.3389/fimmu.2018.01650 30072995 PMC6060442

[142] Thomas G, Micci L, Yang W, et al. Intra-tumoral activation of endosomal TLR pathways reveals a distinct role for TLR3 agonist dependent type-1 interferons in shaping the tumor immune microenvironment. Front Oncol. 2021;11:711673. doi: 10.3389/fonc.2021.711673 34381732 PMC8351420

[143] Hofman L, Lawler SE, Lamfers MLM. The multifaceted role of macrophages in Oncolytic Virotherapy. Viruses. 2021 Aug 9;13(8):1570. doi: 10.3390/v13081570 34452439 PMC8402704

[144] Blitz SE, Kappel AD, Gessler FA, et al. Tumor-associated Macrophages/Microglia in Glioblastoma Oncolytic Virotherapy: a double-edged sword. Int J Mol Sci. 2022 Feb 4;23(3):1808. doi: 10.3390/ijms23031808 35163730 PMC8836356

[145] Martinez FO, Helming L, Gordon S. Alternative activation of macrophages: an immunologic functional perspective. Annu Rev Immunol. 2009;27(1):451–83. doi: 10.1146/annurev.immunol.021908.132532 19105661

[146] Cao F, Nguyen P, Hong B, et al. Engineering Oncolytic vaccinia virus to redirect macrophages to tumor cells. Adv Cell Gene Ther. 2021 Apr;4(2). doi: 10.1002/acg2.99 PMC8021125 33829146

[147] Knox T, Sahakian E, Banik D, et al. Selective HDAC6 inhibitors improve anti-PD-1 immune checkpoint blockade therapy by decreasing the anti-inflammatory phenotype of macrophages and down-regulation of immunosuppressive proteins in tumor cells. Sci Rep. 2019 Apr 16;9(1):6136. doi: 10.1038/s41598-019-42237-3 30992475 PMC6467894

[148] Adams R, Osborn G, Mukhia B, et al. Influencing tumor-associated macrophages in malignant melanoma with monoclonal antibodies. Oncoimmunology. 2022;11(1):2127284. doi: 10.1080/2162402X.2022.2127284 36211808 PMC9543025

[149] Chauhan J, Grandits M, Palhares LCGF, et al. Anti-cancer pro-inflammatory effects of an IgE antibody targeting the melanoma-associated antigen chondroitin sulfate proteoglycan 4. Nat Commun. 2023 Apr 25;14(1):2192. doi: 10.1038/s41467-023-37811-3 37185332 PMC10130092

[150] Williams IP, Crescioli S, Sow HS, et al. In vivo safety profile of a CSPG4-directed IgE antibody in an immunocompetent rat model. MAbs. 2020;12(1):1685349. doi: 10.1080/19420862.2019.1685349 31769737 PMC6927758

[151] Miliotou AN, Papadopoulou LC. CAR T-cell therapy: a new era in cancer immunotherapy. Curr Pharm Biotechnol. 2018;19(1):5–18. doi: 10.2174/1389201019666180418095526 29667553

[152] Soltantoyeh T, Akbari B, Karimi A, et al. Chimeric Antigen Receptor (CAR) T cell therapy for metastatic melanoma: challenges and road ahead. Cells. 2021 Jun 9;10(6):1450. doi: 10.3390/cells10061450 34207884 PMC8230324

[153] Rodriguez-Garcia A, Lynn RC, Poussin M, et al. CAR-T cell-mediated depletion of immunosuppressive tumor-associated macrophages promotes endogenous antitumor immunity and augments adoptive immunotherapy. Nat Commun. 2021 Feb 9;12(1):877. doi: 10.1038/s41467-021-20893-2 33563975 PMC7873057

[154] Wang S, Yang Y, Ma P, et al. CAR-macrophage: An extensive immune enhancer to fight cancer. EBioMedicine. 2022 Feb;76:103873. doi: 10.1016/j.ebiom.2022.103873 35152151 PMC8844597

[155] Klichinsky M, Ruella M, Shestova O, et al. Human chimeric antigen receptor macrophages for cancer immunotherapy. Nat Biotechnol. 2020 Aug;38(8):947–953. 32361713 10.1038/s41587-020-0462-y PMC7883632

[156] Sloas C, Gill S, Klichinsky M. Engineered CAR-Macrophages as adoptive immunotherapies for solid tumors. Front Immunol. 2021;12:783305. doi: 10.3389/fimmu.2021.783305 34899748 PMC8652144

[157] Adams R, Coumbe JEM, Coumbe BGT, et al. BRAF inhibitors and their immunological effects in malignant melanoma. Expert Rev Clin Immunol. 2022 Apr;18(4):347–362. 35195495 10.1080/1744666X.2022.2044796

[158] Atkins MB, Lee SJ, Chmielowski B, et al. Combination dabrafenib and trametinib versus combination nivolumab and ipilimumab for patients with advanced BRAF-Mutant melanoma: the DREAMseq trial—ECOG-ACRIN EA6134. J Clin Oncol. 2023 Jan 10;41(2):186–197. doi: 10.1200/JCO.22.01763 36166727 PMC9839305

[159] Donadon M, Torzilli G, Cortese N. et al. Macrophage morphology correlates with single-cell diversity and prognosis in colorectal liver metastasis. J Exp Med. 2020 Nov 2;217(11). doi: 10.1084/jem.20191847 PMC7596819 32785653

